# Quantitative proteomics analysis of triple-negative breast cancers

**DOI:** 10.1038/s41698-025-00907-8

**Published:** 2025-04-24

**Authors:** Natasha C. Mariano, Jonathan D. Marotti, Youdinghuan Chen, Barbara Karakyriakou, Roberto Salgado, Brock C. Christensen, Todd W. Miller, Arminja N. Kettenbach

**Affiliations:** 1 Department of Biochemistry and Cell Biology, Hanover, NH USA; 2 Department of Pathology and Laboratory Medicine, Lebanon, NH USA; 3 Dartmouth Cancer Center, Lebanon, NH USA; 4 Department of Molecular and Systems Biology, Lebanon, NH USA; 5 Department of Pathology, GZA-ZNA Hospitals, Antwerp, Belgium; 6 Division of Research, Peter MacCallum Cancer Centre, Melbourne, Australia; 7 Department of Epidemiology, Lebanon, NH USA; 8 Department of Community and Family Medicine, Lebanon, NH USA; 9 Department of Pharmacology & Toxicology, Medical College of Wisconsin, Milwaukee, WI USA; 10 Department of Pathology, Medical College of Wisconsin, Milwaukee, WI USA

**Keywords:** Genomic analysis, Proteomic analysis, Breast cancer, Breast cancer

## Abstract

Triple-negative breast cancer (TNBC) accounts for approximately 15% of all Breast Cancer (BC) cases with poorer prognosis and clinical outcomes compared to other BC subtypes due to greater tumor heterogeneity and few therapeutically targetable oncogenic drivers. To reveal actionable pathways for anti-cancer treatment, we use a proteomic approach to quantitatively compare the abundances of 6306 proteins across 55 formalin-fixed and paraffin-embedded (FFPE) TNBC tumors. We identified four major TNBC clusters by unsupervised clustering analysis of protein abundances. Analyses of clinicopathological characteristics revealed associations between the proteomic profiles and clinical phenotypes exhibited by each subtype. We validate the findings by inferring immune and stromal cell type composition from genome-wide DNA methylation profiles. Finally, quantitative proteomics on TNBC cell lines was conducted to identify in vitro models for each subtype. Collectively, our data provide subtype-specific insights into molecular drivers, clinicopathological phenotypes, tumor microenvironment (TME) compositions, and potential pharmacologic vulnerabilities for further investigations.

## Introduction

Breast cancer (BC) remains the second-leading cause of cancer-related death among women in the United States^1^. The triple-negative breast cancer (TNBC) subtype is clinically defined by the absence of estrogen receptor (ER) and progesterone receptor (PR) expression and overexpression/amplification of human epidermal growth factor receptor 2 (HER2)^2^. TNBC accounts for approximately 15% of all BC cases and is considered the most aggressive subtype, with low 5-year survival rates and higher rates of recurrence and metastasis compared to receptor-positive BC^3,4^. The poorer prognosis and clinical outcomes of TNBC are mainly due to a greater heterogeneity among tumors and their microenvironments and a scarcity of therapeutically targetable oncogenic drivers for most TNBC tumors.

Apart from a few recent approvals of targeted therapies for TNBC, predominantly in the metastatic setting, the lack of therapeutic options limits patients to conventional chemotherapy as the primary standard of care treatment^5–10^. The most common chemotherapy regimen for early-stage TNBC involves a combination of anthracyclines (A) and cyclophosphamide (C), followed by taxanes (T), referred to as AC-T^11–13^, which achieves pathological complete response (pCR) rates of 25–50%^14–18^. This limited efficacy, the cytotoxic side effects, and acquired resistance highlight the need for new targeted therapeutic strategies.

Currently, the only targeted therapy outside of the metastatic or advanced setting of TNBC is the immune checkpoint inhibitor pembrolizumab in combination with chemotherapy, which received Federal Drug Administration (FDA) approval for high-risk, early-stage TNBC patients^5,19^. Pembrolizumab blocks the interaction between the programmed death-ligand 1 (PD-L1) surface ligand on tumor cells and the programmed cell death protein 1 (PD-1) inhibitory receptor on T cells. This blockade prevents the functional inactivation or exhaustion of T cells that would otherwise occur when PD-1 and PD-L1 interact, thereby counteracting immune evasion by cancer cells^20–24^. Pembrolizumab was approved for patients whose tumors expressed PD-L1 on their surface. However, this criterion severely limits the number of eligible patients, as only a minority of TNBCs exhibit tumor PD-L1 expression^25–28^. Moreover, pembrolizumab has only achieved partial success in the clinic, even among patients with tumors displaying PD-L1 expression. This observation not only suggests that PD-L1 is a suboptimal biomarker to predict patient response to pembrolizumab but also that the suppressive signals of the PD-1 pathway are not solely responsible for the attenuated immune response in the tumor microenvironment (TME)^25,29^. Hence, a deeper understanding of the interplay between TNBC tumors and their TME is imperative for developing more precise biomarkers and effective therapies.

Numerous studies have been undertaken to identify clinically actionable TNBC subtypes in a comprehensive and unbiased manner using genomic, transcriptomic, and proteomic approaches^30–41^. Given that the output of genomic amplifications is dampened at the protein level and the correlation between transcript numbers to protein levels is often low^42,43^, genomic approaches may not fully capture signaling alterations. Thus, protein-level analyses provide a more comprehensive representation of deregulated signaling and the identification of precision therapeutic strategies.

Here, we use a proteomic approach to quantitatively compare the abundances of 6306 proteins across 55 formalin-fixed and paraffin-embedded (FFPE) TNBC tumors to reveal actionable pathways for anti-cancer treatment. We identified four major TNBC clusters through unsupervised clustering analysis of protein abundances. Following functional annotation analyses of the differentially enriched proteins in each cluster, protein-based subtypes were defined as Immune-activated (IMA), Immune-suppressed (IMS), Luminal androgen receptor (LAR), and Mesenchymal (MES). In addition, analyses of clinicopathological traits revealed associations between proteomic profiles and clinical phenotypes exhibited by each subtype. Furthermore, by inferring immune and stromal cell type composition from genome-wide DNA methylation profiles, we validated the findings of our proteomics analysis. Finally, quantitative proteomics on TNBC cell lines was conducted to identify representative in vitro models for each subtype. Collectively, our analyses provide novel subtype-specific insights such as potential biomarkers, molecular drivers, and pharmacologic vulnerabilities for further investigation.

## Results

### Proteomic analysis of FFPE TNBC tumors

**Fig. 1:**
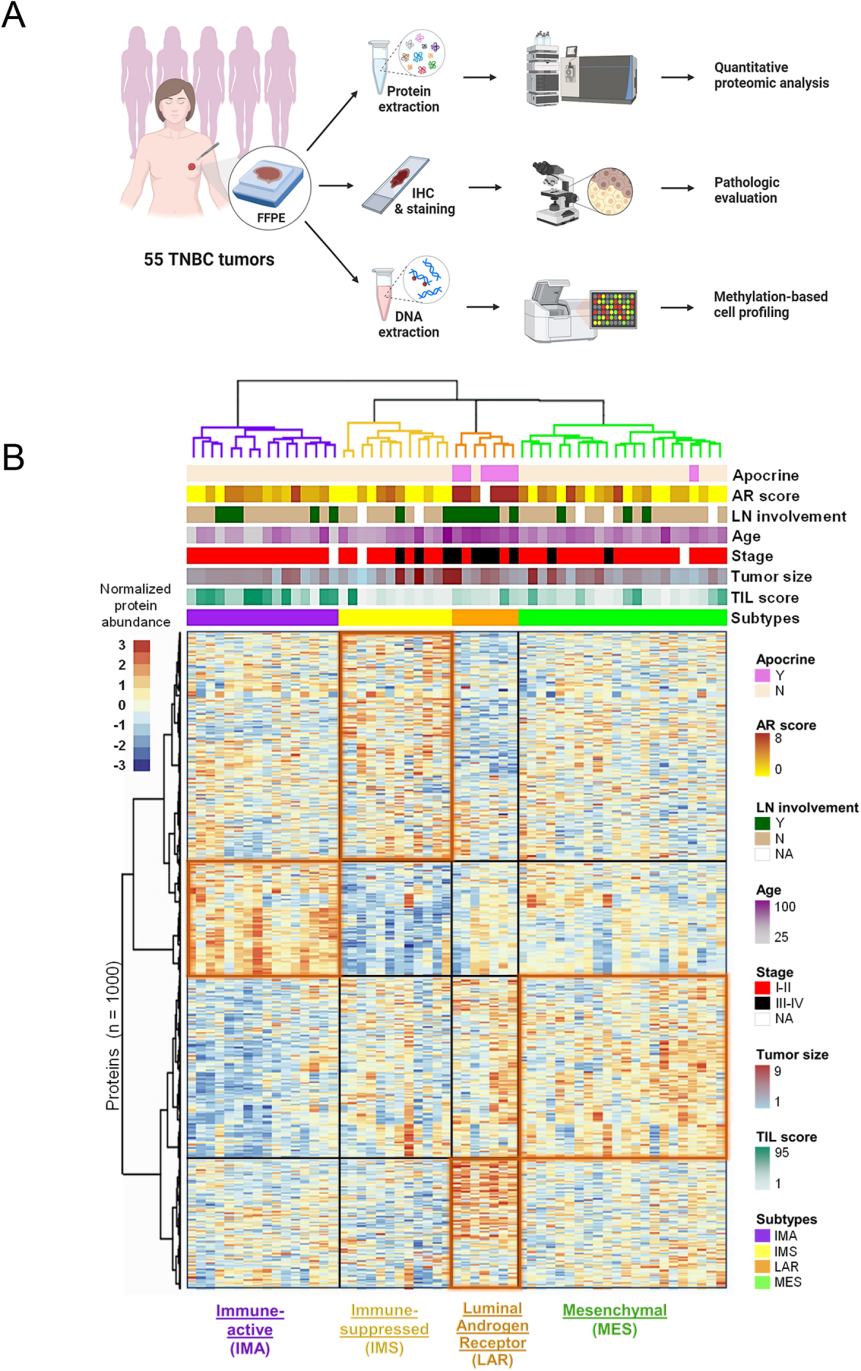
Identification of TNBC subtypes. **A** Overview of the experimental workflow. The quantitative proteomic analysis (top branch) was performed on all 55 tumor specimens (plus two technical replicates), pathologic evaluation (middle branch) was performed on all 55 tumor specimens, and methylation-based cell profiling (bottom branch) was performed on 50 of the tumor specimens. Image created with BioRender.com. **B** Annotated heatmap displaying unsupervised hierarchical clustering analysis of 57 tumor samples (including two technical replicates), which identified four clusters using the relative abundances of the top 1000 most variable proteins based on variance from the mean. Rows represent proteins, and columns represent tumor samples. Annotation tracks are arranged by proteomic subtype (bottom track) and provide a visual comparison of key tumor and patient characteristics by subtype. IHC, immunohistochemistry; FFPE, formalin-fixed paraffin-embedded; Apocrine, apocrine histology presence; AR score, Allred score for androgen receptor staining positivity; LN involvement, cancer-positive lymph nodes detected; Age, patient’s age at diagnosis (in years); Stage, early (I-II) versus late (III-IV) stage cancer at diagnosis; Size, tumor size (in cm); TIL score, tumor-infiltrating lymphocyte percentage; Subtypes, protein-based TNBC subtypes; Y, yes; N, no; NA, missing data (depicted as white space).

We performed quantitative proteomic analysis, pathologic evaluation, and methylation-based cell profiling on 55 primary TNBC tumor specimens at Dartmouth-Hitchcock Medical Center (DHMC) (Fig. 1A). All patients were female, with an average age of 54 years (SD ± 12.8), who underwent surgery between 2003 and 2015 (Table 1). Genetic ancestry estimation using single nucleotide polymorphism (SNP) probes identified most patients as Caucasian, consistent with the ancestry of patients in the DHMC catchment area of New Hampshire and Vermont. The ancestry of six patients could not be determined, potentially due to multiple ancestries. All specimens were classified as triple-negative based on immunohistochemistry (IHC) and fluorescence in situ hybridization (FISH) analysis of ER/PR and HER2/ERBB2, respectively. Tumors were predominantly stage I–II (76%), Scarff-Bloom-Richardson (SBR) grade 9, and treatment-naïve. Histopathological features and clinical characteristics are summarized in Table 1. For proteomic analysis, sections from FFPE tumor tissue were macro-dissected if needed to enrich for regions with malignant cancer cells (≥70% malignant cellularity). FFPE fixation was reversed, and protein was extracted and digested into peptides. Samples were randomly assigned a unique tandem mass tag (TMT) and multiplexed and offline-fractionated before LC-MS^3^ analysis. A frequency of observation cutoff of 93% was implemented, resulting in the identification and quantification of 6306 proteins (Supplementary Table 1).

**Table 1:** Clinical characteristics of TNBC cohor

No. of patients	55	(100%)
**Age at diagnosis**		
<45	11	(20%)
45–65	34	(62%)
>65	10	(18%)
**Apocrine**		
Positive	7	(13%)
Negative	48	(87%)
**AR score**		
<3	24	(44%)
3–5	20	(36%)
>5	9	(16%)
Missing	2	(4%)
**Stage at diagnosis**		
I-II	42	(76%)
III–IV	10	(18%)
Missing	3	(5%)
**Tumor size**		
≤3	25	(45%)
3-5	23	(42%)
>5	6	(11%)
Missing	1	(2%)
**LN involvement**		
Positive	15	(27%)
Negative	35	(64%)
Missing	5	(9%)
**S.B.R score**		
Grade 9	46	(84%)
Grade ≤8	9	(16%)
**Associated DCIS**		
Positive	36	(65%)
Negative	18	(33%)
Missing	1	(2%)
**LV invasion**		
Positive	23	(42%)
Negative	31	(56%)
Missing	1	(2%)
(**TIL score**		
<15	21	(38%)
16–60	25	(45%)
>60	9	(16%)
**BRCA-like**		
Positive	46	(84%)
Negative	9	(16%)
**Fibrosis**		
Positive	10	(18%)
Negative	45	(82%)
**Infiltrating margins**		
Positive	43	(78%)
Negative	12	(22%)

### Functional annotation of TNBC protein-based subtypes

**Fig. 2:**
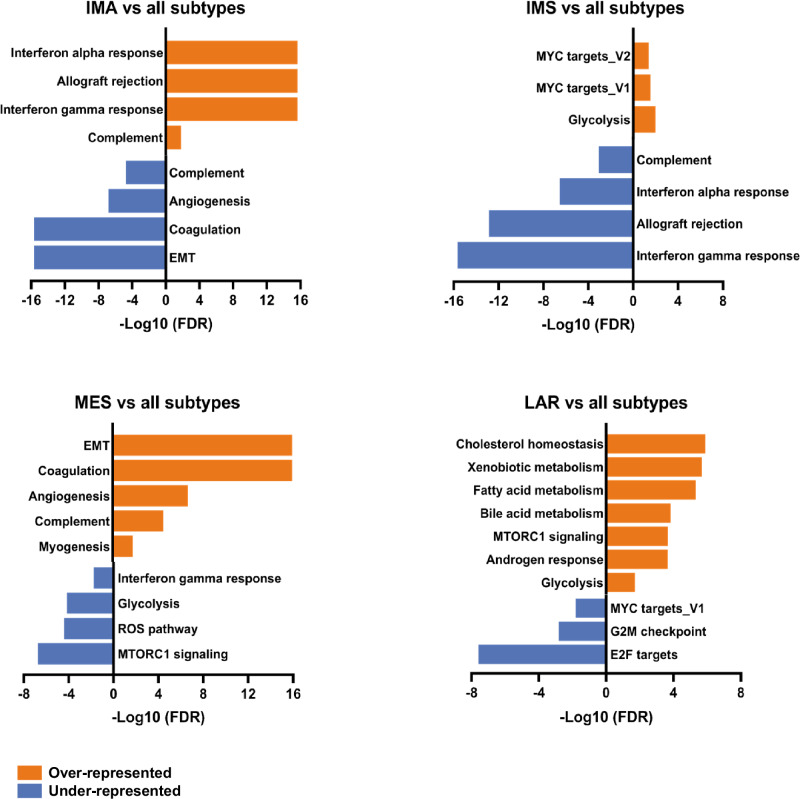
Hallmark gene set enrichments by TNBC subtype. Bar plots showing over-representation analysis (ORA) results for enriched Hallmark gene sets in each protein-based TNBC subtype, using their 200 highest and lowest DAPs as input into WebGestalt. A Benjamini–Hochberg adjusted p-value (FDR) <0.05 was considered statistically significant for functional annotation enrichment. All significant results are shown and were plotted using the −Log_10_(FDR) value. Terms to the right of the *y*-axis (orange bars) indicate over-represented Hallmarks, while the terms to the left of the *y*-axis (blue bars) indicate the under-represented Hallmarks and were given a negative sign in front of their −Log_10_(FDR) values to signify/visualize low enrichment in the bar plot.

**Fig. 3:**
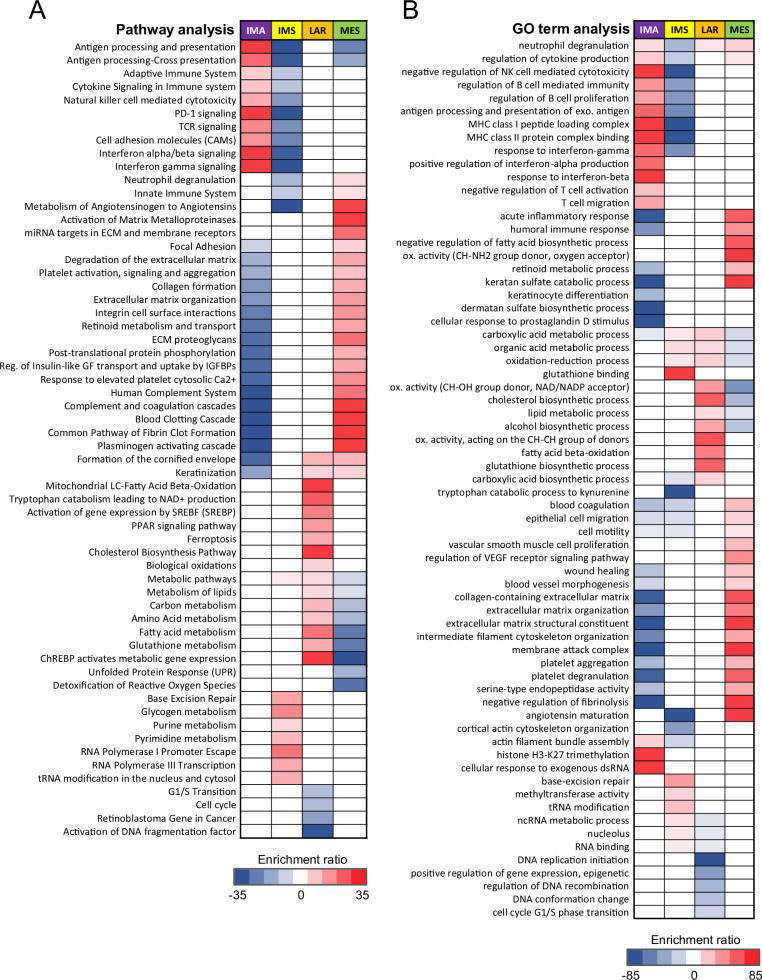
Functional annotation heatmaps. The heatmaps depict representative ORA results from Supplementary Table 2 for (**A**) molecular pathways (from KEGG, Reactome, Panther, Wikipathways databases) and (**B**) gene ontology (GO) terms in each TNBC subtype, using the subtype-specific DAPs as input. Enrichment ratios derived from the ORA were used to signify the association level between the terms and TNBC subtypes. Under-represented terms were given a negative sign before their enrichment ratios and colored blue to indicate the lowly enriched terms, while the highly enriched terms were colored red. A white box color indicates no association. KEGG, Kyoto Encyclopedia of Genes and Genomes; exo, exogenous.

To identify distinct protein-based subtypes within the TNBC tumor cohort, we performed unsupervised hierarchical clustering on the top 1000 most variable proteins in the dataset. Four robustly segregated groups were identified based on their separation in the heatmap dendrogram (Fig. 1B) and their distinct distribution in a principal component analysis (PCA) plot (Supplementary Fig. 1A). Moreover, to independently ensure the stability of the subtype classifications, we employed k-means consensus clustering on the tumor proteomic dataset. We determined the optimal number of clusters based on the point at which the cumulative distribution function (CDF) area ceases to show a corresponding marked increase with a further increase in cluster number. (Supplementary. Fig. 1B, C). To identify actionable protein-based oncogenic changes for each of the four clusters, we performed functional annotation analyses. Differential protein analysis was carried out for each subtype, identifying the top proteins with significant increases and decreases in abundance (Supplementary Table 2). Using these proteins, we conducted over- and under-representation functional annotation analyses (Supplementary Table 2) to identify enriched Hallmark gene sets (Fig. 2), gene ontologies (GO), and biological pathways (Fig. 3)^44^. Based on these results, we will refer to the four clusters as the immune-active (IMA, purple), immune-suppressed (IMS, yellow), luminal androgen receptor (LAR, orange), and mesenchymal (MES, green) subtypes (Fig. 1B).

The most distinct TNBC tumor cluster was the IMA subtype (Fig. 1B), characterized by a strong over-representation of immune-related functional annotations. The most significantly enriched Hallmarks in IMA tumors included Interferon alpha (IFN-α) response and Interferon gamma (IFN-γ) response. Concurrently, the GO analysis revealed similar terms, such as regulation of cytokine production, B cell-mediated immunity, and involvement of MHC class I and II complexes. Pathway analysis further highlighted immune-related signatures with enrichment in PD-1 signaling, T cell receptor (TCR) signaling, and Natural killer (NK) cell-mediated cytotoxicity. Collectively, these annotations suggest a high infiltration of cytotoxic and helper T lymphocytes, as well as NK cells and B cells in the tumor environment belonging to this subtype. Moreover, the various terms involving antigen presentation imply the presence of antigen-presenting cells, such as dendritic cells (DCs), which typically activate T cells under normal conditions or dysregulate T cells under cancerous conditions^45,46^.

Conversely, the annotations notably under-represented in the IMA subtype were predominantly stroma-associated. Hallmarks analysis revealed a decrease in the terms Epithelial-to-mesenchymal transition (EMT), Coagulation, and Angiogenesis. Likewise, the GO terms showed collagen-containing extracellular matrix, extracellular matrix organization, serine-type endopeptidase activity, epithelial cell migration, and wound healing. Combined, these analyses suggest that the TME of IMA tumors exhibit reduced extracellular matrix (ECM) remodeling, EMT, and angiogenic processes compared to the other subtypes. The reduction in angiogenesis is consistent with the high IFN-γ signaling observed in this subtype since IFN-γ is an anti-angiogenic cytokine^47^. Moreover, under-representation of the blood clotting cascade, platelet signaling, fibrin clot formation, and response to elevated platelet cytosolic Ca^2+^ pathways indicate relatively low levels of infiltration by platelets or other stromal cells able to deposit and rearrange ECM components.

The over-represented functional annotations for the IMS subtype consisted of DNA repair-, RNA-, and metabolism-related signatures. The most significantly enriched Hallmarks included MYC variant target genes and Glycolysis. Similarly, GO terms were enriched in carboxylic acid metabolic, organic acid metabolic, ncRNA metabolic, and oxidation-reduction processes. Pathway analysis revealed enrichment in base excision repair, tRNA modification, and RNA Polymerase I and III activity. While DNA repair defects and metabolic dysregulation are already well-established hallmarks of numerous cancer types, the RNA-related signature of the IMS subtype, which likely signifies tumorigenic alterations to fundamental RNA-mediated processes, provides new avenues to explore potential subtype-specific resistance mechanisms and cancer vulnerabilities. Moreover, unlike studies that profile the RNA species involved in TNBC pathogenesis, our investigation sheds light on proteins involved in abnormal functions^37,48–50^. Intriguingly, the under-represented immune-related functional annotations were the most prominent features of the IMS subtype. For the Hallmarks analysis, IFN-α and IFN-γ responses were under-represented. In the GO analysis, TCR signaling, cytokine signaling, antigen presentation, NK cell-mediated cytotoxicity, and B cell proliferation pathways were under-represented, as well as pathways indicating reduced innate and adaptive immune system presence. These results hint that in IMS tumors, immune cell types such as T lymphocytes (cytotoxic and helper), B lymphocytes, DCs, neutrophils, and NK cells are depleted in the TME.

The LAR subtype revealed distinctive functional annotations characterized by the over-representation of metabolism-related signatures. The most significant Hallmarks included Cholesterol homeostasis, Fatty acid metabolism, MTORC1 signaling, and Androgen response. GO analysis demonstrated similar metabolic terms, including fatty acid beta-oxidation, carboxylic acid biosynthetic process, cellular modified amino acid metabolic process, and oxidoreductase activity with NAD/NADP as an acceptor. In addition to the general ‘Metabolic pathways’ term, pathway analysis revealed potential signaling cascade targets such as the PPAR pathway, activation of gene expression by SREBF (SREBP), biological oxidations, and tryptophan catabolism leading to NAD+ production. The predominant metabolism-related signature of the LAR subtype suggests that tumors have undergone metabolic reprogramming with unconventional mechanisms to mediate hyperactive energy consumption. Moreover, the oxidation-reduction processes associated with alternative energy utilization may indicate increased levels of reactive oxygen species (ROS), which can act as immunomodulators in the TME^51–55^. The LAR subtype was also enriched in keratinization, which suggests a more differentiated and less mesenchymal phenotype^56^. The under-represented functional annotations for the LAR subtype were predominantly RNA-, DNA- and cell cycle-associated signatures. The most significant Hallmarks were E2F targets, G2M checkpoint, and MYC variant targets, accompanied by the GO terms RNA binding, ncRNA metabolic process, DNA recombination, DNA conformation change, and regulation of epigenetic gene expression. As for under-represented pathways, they included cell cycle, G1/S transition, and Retinoblastoma gene in cancer. The reduced cell cycle and proliferation signatures uphold the observation that the androgen-driven TNBC tumors are known to be slower-growing tumors with fewer Ki67-positive cells^57,58^.

Lastly, the MES cluster displayed predominant enrichment in stromal- and immune-related signatures. The most significant Hallmarks included Complement, Angiogenesis, EMT, and Coagulation. Similarly, the GO analysis revealed epithelial cell migration, intermediate filament cytoskeleton organization, serine-type endopeptidase activity, collagen-containing extracellular matrix, VEGF signaling, and wound healing. In addition, platelet signaling, response to elevated platelet cytosolic Ca^2+^, degradation of ECM, collagen formation, ECM proteoglycans, miRNA targets in ECM, and activation of matrix metalloproteinases (MMPs) pathways were significantly enriched. Notably, the MES subtype also showed enrichment of immune signatures related to inflammatory, innate, and humoral immune responses. Indeed, enrichment of neutrophil degranulation, regulation of cytokine production, acute inflammatory response, humoral immune response, and human complement system suggests a different immune microenvironment than that of the IMA subtype, including the possible conversion of helper T cells to a tumor-promoting (Th-2) phenotype^59^. Furthermore, terms indicating the re-organization of the ECM suggest highly active and dynamic TME components. In particular, the deposition of several upregulated ECM proteins, such as fibronectin (FN1) and collagen, into the MES subtype’s TME may act as a stiff barrier that blocks immune cells from infiltrating the tumor and generating effective anti-tumor immunity^60–62^.

The MES subtype showed an under-representation of functional annotations that were predominantly metabolism-associated. The under-represented Hallmarks consisted of MTORC1 signaling, Glycolysis, and ROS pathway, accompanied by the GO terms oxidation-reduction process, and organic acid-, carboxylic acid-, and lipid- metabolic processes. In addition, the significantly under-represented pathways included metabolic pathways, unfolded protein response, and detoxification of ROS. The reduced metabolic activity in this subtype could suggest that MES tumors are not as focused on primary tumor growth or bulk tumor cell proliferation as other subtypes. Instead, the MES tumors appear to prioritize mechanisms of invasion and metastasis, with its hyperactive stromal and immune TME driving its rapid cancer progression.

To validate these findings, we analyzed an independent TNBC quantitative proteomics dataset published by Anurag et al.^35^ A comparison of both datasets revealed that 886 of the 1000 most variable proteins in our dataset were also identified and quantified in the Anurag et al. dataset. Unsupervised hierarchical clustering of the relative protein abundances of the 886 proteins largely recapitulated the four subtypes identified in our original analysis (Supplementary Fig. 2). Next, we performed unsupervised hierarchical clustering and differential protein analysis on the Anurag et al.^35^ dataset, which identified four subtypes with enrichment of Hallmark gene sets similar to those observed in our dataset, further validating our approach and subtype annotation (Supplementary Fig. 3A, B, Supplementary Table 3). A limitation of this comparison is that the Anurag et al. cohort consisted of Stage 2 and Stage 3 tumors. In contrast, 30% of tumor samples in our analysis were classified as Stage 1 and Stage 4. For instance, we found that the IMA subtype exclusively contained early-stage tumors (Stages 1 and 2), while the LAR subtype contained late-stage tumors (Stages 3 and 4). Despite these differences, we were able to identify a cluster in the Anurag dataset that recapitulated the IMA subtype, featuring upregulated immune signatures and downregulated EMT/stromal signatures, and another cluster that resembled the LAR subtype, with upregulated metabolic signatures and downregulated proliferation signatures.

### Clinicopathological analysis of TNBC tumor specimens

We conducted histopathological assessments of tumors to investigate potential associations with proteomic TNBC subtypes (Table 1). Among the subtypes, IMA exhibited the highest proportion of tumors with pushing borders (71%) and was exclusively comprised of primary tumors from patients with early-stage disease (100%). Consistent with the observed angiogenesis and ECM remodeling signatures from the under-represented functional annotation analyses (Figs. 2 and 3), the IMA subtype demonstrated the lowest incidence of tumors with lymphovascular invasion (31%) (Supplementary Fig. 4B); this may indicate relatively early steps in tumor development and/or suggest the active immune system within the IMA TME may contribute to tumor containment.

**Fig. 4:**
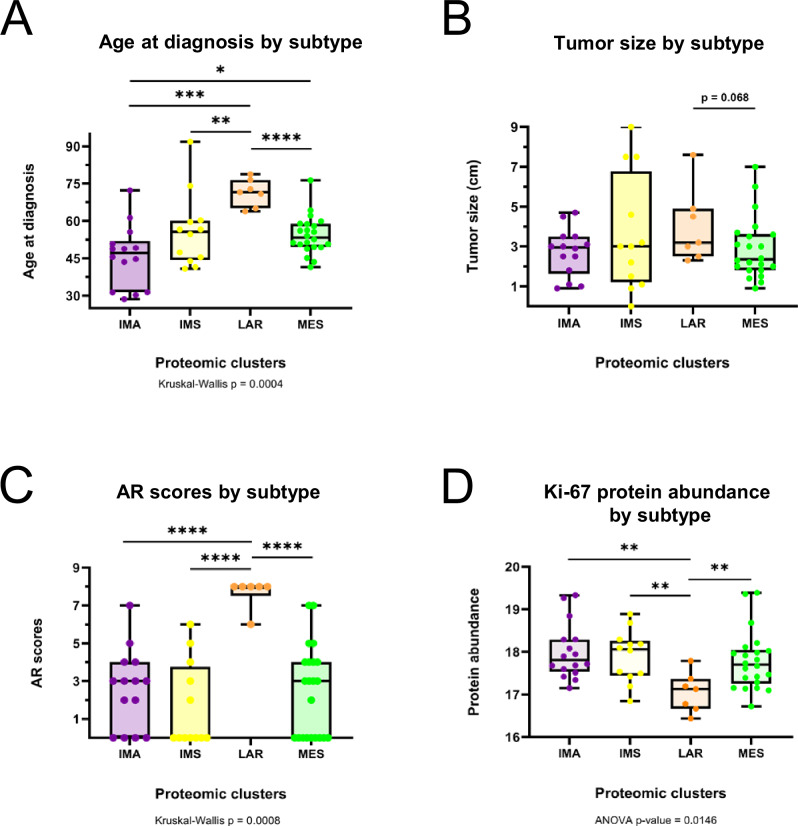
Clinicopathological features associated with TNBC subtypes. Boxplots showing the distribution of (**A**) patients’ age at the time of diagnosis, (**B**) tumor sizes (in cm), (**C**) androgen receptor staining using Allred scoring, and (**D**) Ki67 protein abundances across the four proteomic TNBC subtypes. **A–D** The middle bar represents the median value and the box edges represent the interquartile range. Either one-way analysis of variance (ANOVA) or Kruskal–Wallace was used for statistical testing. For pairwise comparisons between subtypes, unpaired two-tailed Welch’s *t* test was performed, and a statistically significant *p*-value is shown as (*p <0.05), (**p <0.01), (***p <0.001), (****p <0.0001).

The LAR subtype exhibited the oldest age at diagnosis, the largest average tumor size, and the highest proportion of patients with late-stage disease (71%) (Fig. 4A, B, Supplementary Fig. 4C). As expected, the LAR tumors displayed prominent apocrine differentiation with six out of seven apocrine tumors falling within this cluster (Supplementary Fig. 4D), as well as the highest Allred scores for androgen receptor (AR) staining (score of 8) in six out of seven tumors (Fig. 4C). Furthermore, the LAR subtype presented the highest proportion of tumors with lymphovascular invasion (86%) (Supplementary Fig. 4B), infiltrating margins (100%) (Supplementary Fig. 4E), and positive lymph nodes (86%) (Supplementary Fig. 4F), plus a lack of pushing borders (Supplementary Fig. 4A). Conversely, LAR tumors had the lowest abundance of Ki67 protein (Fig. 4D) and the lowest proportion of SBR grade 9 (43%) (Supplementary Fig. 4G)^63^. Lastly, LAR tumors had a relatively high degree of prominent fibrosis (29%) ( >50% of the tumor stroma is fibrotic) and were all associated with DCIS (100%) (Supplementary Fig. 4H, I).

IMS subtype tumors were the second-largest in size overall (Fig. 4B), had the highest Ki67 protein abundance (Fig. 4D), and exhibited a relatively high proportion of tumors with the maximum SBR grade of 9 (92%) (Supplementary Fig. 4G), consistent with the IMS subtype’s functional annotations suggesting one of the highest proliferative rates among the subtypes (Figs. 2 and 3). Meanwhile, this subtype exhibited the lowest Allred scores for AR staining (Fig. 4C). The IMS subtype displayed the second-greatest proportion of tumors with pushing borders (58%), lymphovascular invasion (50%), late-stage disease (27%), and the lowest incidence of infiltrating margins (58%) (Supplementary Fig. 4A–C, E).

MES subtype tumors consisted predominantly of early-stage cancer cases (90%) with a high proportion of infiltrating margins (86%) and the lowest proportion of positive lymph nodes among the subtypes (16%) (Supplementary Fig. 4C, E, F). The MES subtype also showed the greatest proportion of cases with prominent fibrosis (36%), accounting for 8 of the 10 reported fibrotic tumors (Supplementary Fig. 4H), in agreement with the enrichment of terms associated with ECM remodeling and collagen deposition observed in the functional annotation analysis (Fig. 3).

**Fig. 5:**
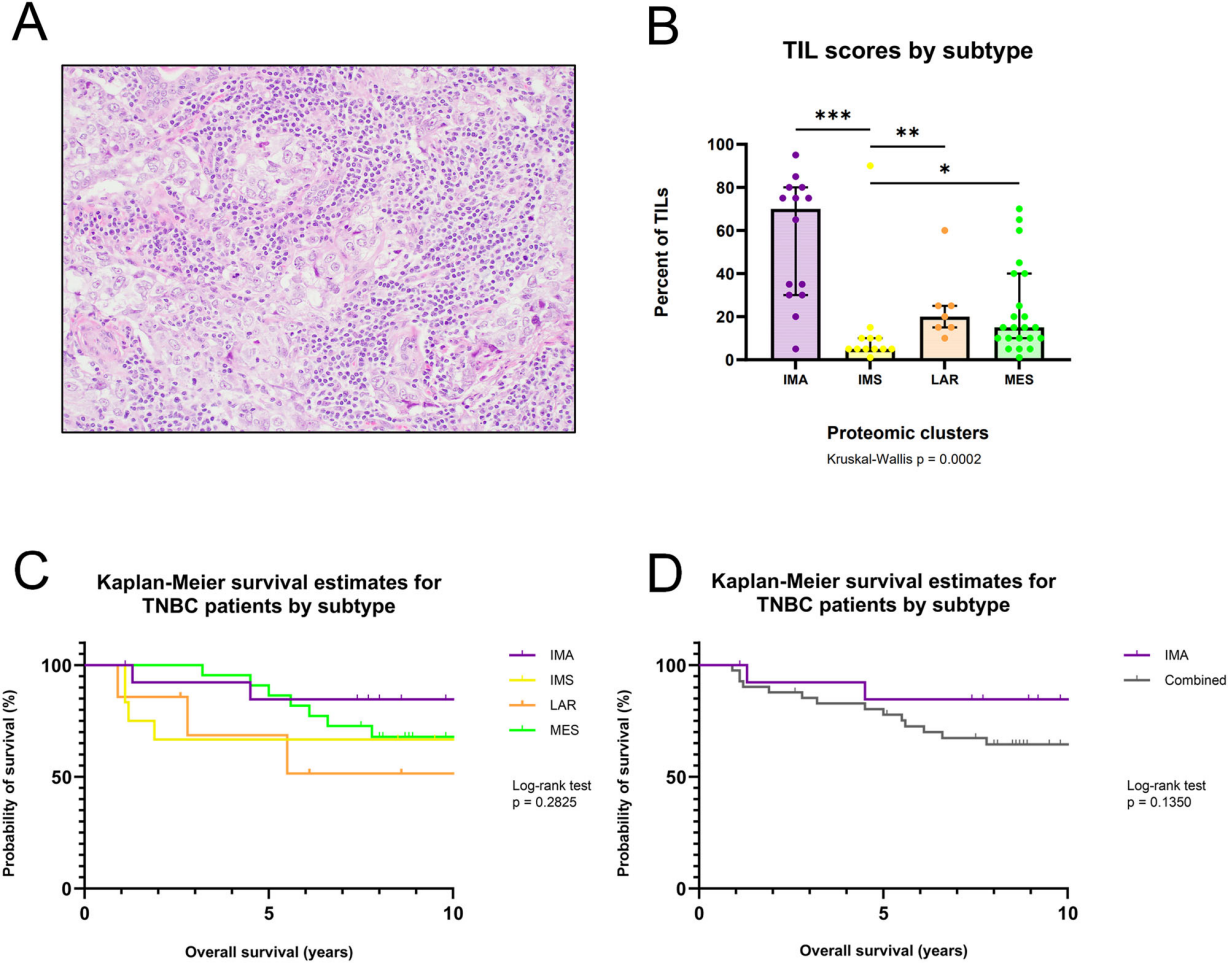
Immune-related clinical features by TNBC subtype. **A** Representative image of tumor-infiltrating lymphocytes (TILs) in a TNBC tumor section using hematoxylin and eosin (H&E) staining. **B** Bar plot comparing the percentage distribution of TILs between the four proteomic TNBC subtypes. The bar height represents the median percentage, with error bars that extend to the interquartile range. Each data point is one case. Asterisks show the pairwise significance (*p <0.05), (**p <0.01), (***p <0.001), derived from an unpaired two-tailed Welch’s *t* test. Kaplan–Meier plots comparing overall survival (OS) outcomes of patients during a 10-year follow-up time (**C**) between all four proteomic subtypes and (**D**) between the IMA subtype against the other three subtypes combined. Log-rank test of *p* < 0.05 was used to determine statistical significance.

Proteomics analysis pointed to the differential involvement of immune cells across subtypes. To further investigate this, we assessed the percent distribution of tumor-infiltrating lymphocytes (TILs) across each tumor specimen in the TNBC cohort (Fig. 5A). Consistent with their proteomic signatures, IMA tumors were highly enriched in TILs, while IMS tumors had the lowest TIL counts (Fig. 5B). Since high TIL scores in TNBC tumors portend a relatively good prognosis^64–68^, we performed an overall survival (OS) analysis between subtypes (*p* = 0.283) (Fig. 5C) and a subsequent analysis between the high-TIL IMA subtype vs. the other three subtypes combined (*p* = 0.135) (Fig. 5D). Although IMA had the best OS among the four subtypes, neither of these survival comparisons reached statistical significance, likely due to the small sample size of the cohort.

### Cell-type deconvolution by DNA methylation profiling

Since the TME appeared to be a driving influence in the generation of the protein-based subtypes, we investigated the composition of the tumors and their microenvironments using cell-type deconvolution analysis based on DNA methylation profiles^69^. We applied Illumina HumanMethylationEPIC technology and the HiTIMED algorithm to identify angiogenic cell types (epithelial, endothelial, and stromal) and immune cell types (neutrophils, DCs, B cells, helper (CD4+) T cells, cytotoxic (CD8+) T cells, regulatory T (Treg) cells, and NK cells)^69,70^.

**Fig. 6:**
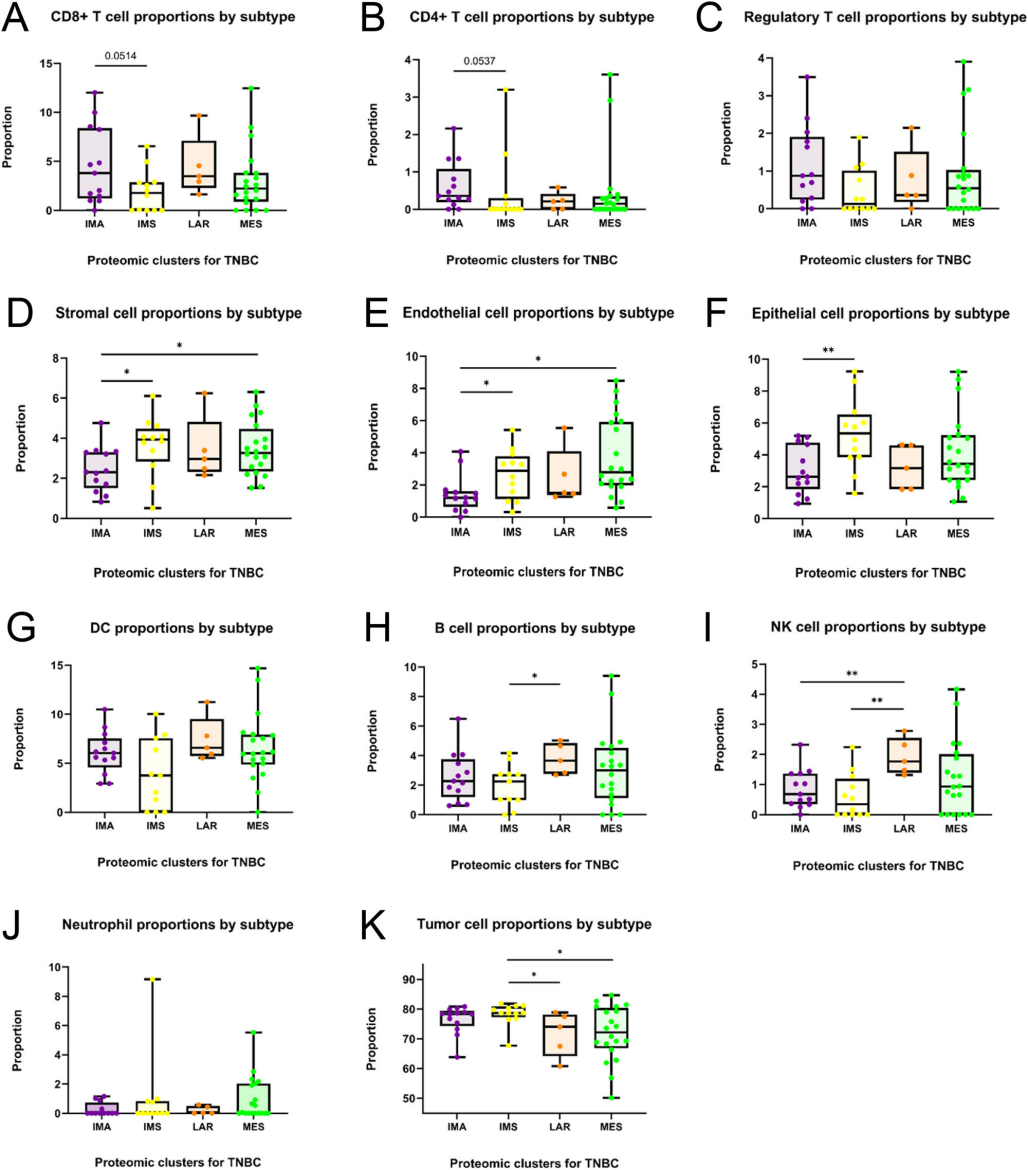
Deconvolution of major cell-types in the TME of each TNBC subtype. Boxplots comparing immune and angiogenic category cell-type proportions: (**A**) CD8+ T lymphocytes, (**B**) CD4+ T lymphocytes, (**C**) Regulatory T lymphocytes (Tregs), (**D**) Stromal cells, (**E**) Endothelial cells, (**F**) Epithelial cells, (**G**) Dendritic cells (DCs), (**H**) B cells, (**I**) Natural-killer (NK) cells, (**J**) Neutrophils, and (**K**) Tumor cells across the four proteomic TNBC subtypes. The middle bar represents the median value, and the box edges represent the interquartile range. Each data point is one case. Asterisks show the pairwise significance from unpaired two-tailed Welch’s *t* test (*p <0.05), (**p <0.01).

Here, the IMA subtype displayed the highest proportions of CD8+ T, CD4+ T, and Treg cells, inferred from cell-type deconvolution analysis (Fig. 6A–C). Furthermore, the IMA subtype exhibited the lowest inferred proportions of angiogenic cell types (Fig. 6D–F), in agreement with the under-representation of stromal- and angiogenic-related functional annotations (Figs. 2 and 3).

In contrast, IMS tumors exhibited the highest inferred proportions of all angiogenic cell types, including stromal, endothelial, and epithelial cells, which reached statistical significance when compared to IMA tumors (Fig. 6D–F), and the lowest inferred proportions for most immune cell types, including CD8 + T, CD4 + T, Treg, DC, B, and NK cells (Figure 6A-c/G-I). The comparably low number of immune cells in the IMS subtype TME aligns with the immune-suppressive signatures observed in the proteomic and TIL score analyses.

The MES subtype showed high inferred proportions of stromal and endothelial cell types but not epithelial cell types (Fig. 6D–F), consistent with its enrichment in stromal-related and EMT functional annotations (Figs. 2 and 3). In addition, the MES subtype’s continued proclivity toward alternative immune responses, such as humoral and innate, was evident in the DNA methylation analysis, as it displayed high inferred proportions of B cells, NK cells, and neutrophils (Fig. 6H–J).

Despite exhibiting primarily metabolism-related functional annotations in the proteomics analysis, LAR tumors had the greatest proportions of DC, B cell, and NK cell types, as well as high levels of CD8+ T cells (Fig. 6A, G–I). Although surprising, these findings might explain the high lymph node involvement and lymphovascular invasion observed in the pathological assessment (Supplementary Fig. 4B, F).

To validate these findings, we used the ProteoMixture analysis tool, a protein-based cell type deconvolution approach on our proteomic dataset^71^. Notably, we identified a similar distribution of stroma, tumor, and immune cell types in each of the four subtypes using this approach as with the DNA methylation-based deconvolution analysis (Supplementary Fig. 5 A–C).

### Proteomic subtyping of TNBC cell lines

To gain insight into the underlying tumor biology of the TNBC protein-based subtypes, we sought to identify cell lines representative of each tumor subtype for pre-clinical studies. We performed quantitative global proteomics analysis on 11 TNBC cell lines. In total, 8518 proteins were quantified across all cell lines, with 8484 proteins quantified in at least seven lines (Supplementary. Table 4). Of the 8484 proteins, 5535 were quantified in the primary tumor dataset, as well (Supplementary Fig. 6B). Unsupervised hierarchical clustering identified three distinct clusters (Clusters 1-3) (Supplementary Fig. 6A).

**Fig. 7:**
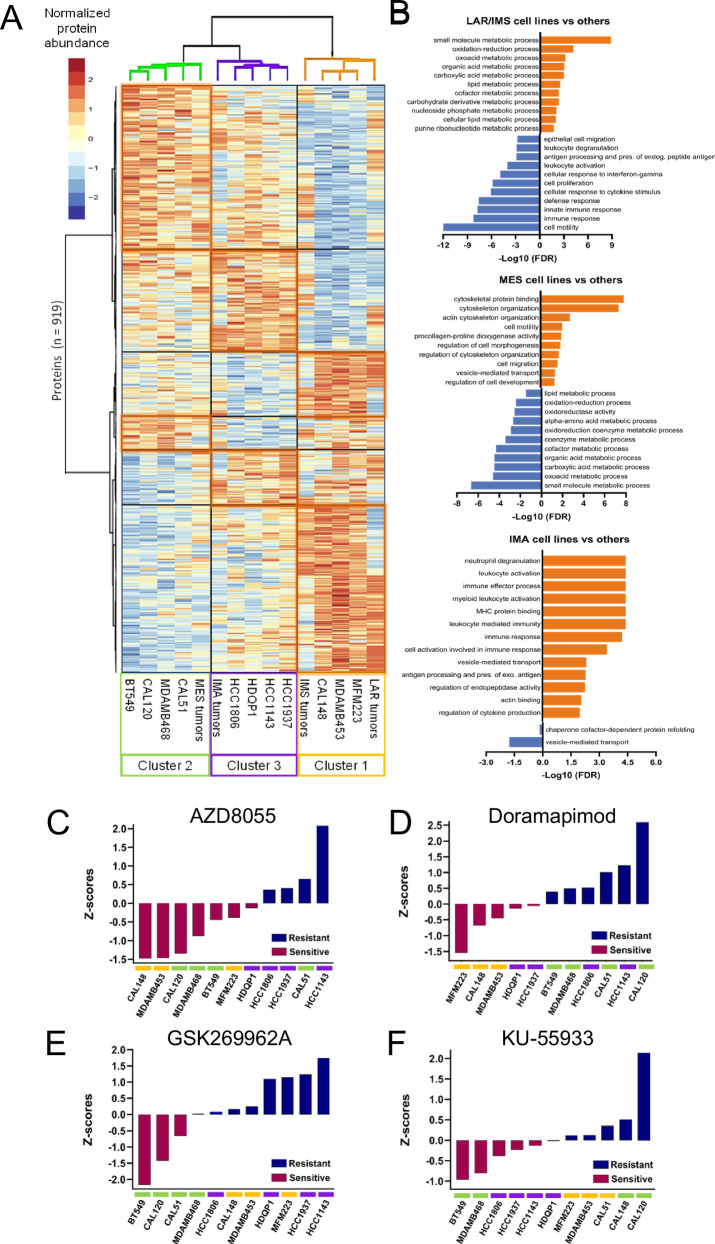
Subtype assignments and differential drug sensitivities of representative TNBC cell lines. **A** Heatmap displaying unsupervised hierarchical clustering of the merged proteomic datasets (11 cell lines and 4 averaged tumor samples) using the DAPs found between cell line Clusters 1-3 alone. **B** Bar plots depicting over-representation analysis using cell line cluster-specific DAPs. The GO terms displayed were selected based on significance (FDR <0.05) and functions shared between cell lines and the tumor subtypes they represent. **C–F** Bar graphs depicting the responses of cell line Clusters 1-3 to pathway inhibitors targeting (**C**) MTORC1 (AZD8055) signaling and (**D**) JNK/P38 (Doramapimod) signaling, (**E**) ROCK/Rho (GSK269962A) signaling, and (**F**) ATM (KU-55933) signaling. The corresponding drug response information for the 11 TNBC cell lines was sourced primarily from the GDSC2 dataset on the Genomics of Drug Sensitivity in Cancer (GDSC) database. Responses are measured by the z-score of half-maximal inhibitory concentration, where a negative z-score (red bar) indicates sensitivity and a positive z-score (purple bar) indicates resistance.

To compare the proteomics profiles of primary TNBC tumors and cell lines, we identified differentially abundant proteins in the cell line dataset and performed unsupervised hierarchical clustering combined with the average protein abundances of the four tumor subtypes from our tumor analysis (Fig. 7A). The results showed that the Cluster 1 cell lines (MFM223, MDAMB453, and CAL148) segregated with both the IMS and LAR subtypes, the Cluster 2 cell lines (BT549, CAL120, CAL51, and MDAMB468) with the MES subtype, and the Cluster 3 cell lines (HCC1937, HCC1143, HCC1806, and HDQP1) with the IMA subtype. This clustering behavior is consistent with previous TNBC cell line subtype assignments published in Lehmann et al.^30^, with the exception of MDAMB468. MDAMB468 was previously assigned to a basal-like subtype, which aligned with our IMA subtype. However, MDAMB468 had the lowest subtype correlation score of cell lines in this subtype (0.19, *p*-value 0.06)^30^.

We then performed GO term analysis on each cell line cluster without the tumor samples (Fig. 7B). The comparison between the cell line functional annotations and tumor signatures revealed that protein-based TNBC tumor subtyping is driven mainly by constituents of the TME, such as stromal and immune response proteins. Despite the absence of a TME in vitro, the cell lines recapitulated many functional annotations observed in their tumor counterparts.

GO annotation analysis of cell line Cluster 1 showed an over-representation of metabolism-related signatures (Fig. 7B) that were also shared with both the LAR and IMS tumor subtypes (Figs. 3B and 7B). These included metabolic and oxidoreductase activities, with functional terms such as carboxylic acid metabolic process, organic acid metabolic process, and oxidation-reduction process. Among the mutually under-represented functional annotations were primarily the immune-related terms shared with the IMS subtype, including leukocyte activation, antigen processing and presentation, response to IFN gamma, and cellular response to cytokine stimulus. Thus, Cluster 1 cell lines can represent LAR and IMS tumor subtypes in pre-clinical studies that seek to test metabolic vulnerabilities, specifically.

GO annotation analysis of Cluster 2 cell lines showed a high enrichment of cell morphogenesis and migration (Fig. 7B). Similarly to the MES tumor signatures, Cluster 2 displayed annotations including cell migration, cell motility, and cytoskeleton organization. The under-represented terms were predominantly related to metabolism and oxidoreductase activity consistent with the MES tumor results (Figs. 3B and 7B). Therefore, Cluster 2 cell lines represent suitable models for studying the MES tumor subtype in pre-clinical studies addressing properties related to EMT and cell migration. However, investigations into ECM remodeling and angiogenesis will likely require in vivo models to capture the full complexity of these processes.

The over-represented GO annotations from Cluster 3 cell lines that were shared with the IMA tumor subtype showed a high enrichment of terms involved in immune response and proliferation, such as leukocyte-mediated immunity, MHC protein binding, antigen processing and presentation, and cytokine production (Figs. 3B and 7B). Notably, there were no significantly under-represented terms shared between the IMA tumor subtype and Cluster 3 cell lines. Therefore, Cluster 3 cell lines can represent the IMA tumor subtype in pre-clinical studies that test aspects of immune signaling and pathways. However, to adequately examine the dynamics and interplay between cancer cells and the immune system, investigations will likely require in vivo models.

Next, we used the Genomics of Drug Sensitivity in Cancer (GDSC) database to identify drug vulnerabilities corresponding to the proteomics-based TNBC tumor subtypes. Consistent with the metabolically hyperactive state of the LAR and IMS subtypes, cell lines in Cluster 1 were particularly sensitive to a mTORC1/2 inhibitor (AZD8055) and a p38/JNK inhibitor (doramapimod) (Fig. 7C/D). Both inhibitors target metabolic processes enriched in the functional annotations of the LAR and IMS subtypes: the mTORC1/2 pathway is a nutrient/energy-sensing pathway activated by pools of intracellular amino acids^72^; the p38/JNK pathway regulates apoptosis activated by oxidative stress^73,74^. Cell lines in Cluster 2, which grouped with the metabolically low MES subtype, were particularly resistant to the p38/JNK inhibitor doramapimod (Fig. 7D) but sensitive to inhibition of the Rho/ROCK pathway (GSK269962A) (Fig. 7E). Rho/ROCK regulates angiogenesis and cell migration by mediating cytoskeleton dynamics^75^, which are processes highly enriched in the MES subtype’s functional annotation analysis. Lastly, the representative IMA subtype cell lines in Cluster 3 were sensitive to drugs that deregulate DNA replication, such as an ATM inhibitor (KU-55933) (Fig. 7F), which mediates apoptosis induced by DNA damage and cell cycle arrest^76^.

## Discussion

TNBC continues to be treated as a single disease with few targeted therapies available and for only a subset of patients, while the response to the standard-of-care chemotherapy regimen is limited for many of the remaining patients. In part, this is due to the heterogeneous nature of TNBC tumors that display varying resistance to the “one-size-fits-all” therapeutic approach. Therefore, it is imperative to identify the molecularly distinct subtypes within TNBC and characterize their unique biology to develop more efficacious and personalized treatment strategies. Despite technological advances, few studies have investigated the proteomic landscapes of TNBC tumors^32,40,41^. In this study, we used quantitative proteomics to profile 55 untreated primary TNBC tumors. We identified four proteomic subtypes enriched in distinct functional annotations, clinicopathological phenotypes, and TME compositions. By combining the quantitative proteomics datasets of TNBC tumors and cell lines, we also identified cell line models representative of each tumor subtype.

Among the four TNBC tumor subtypes, the IMA subtype features a strong immune response signature accompanied by reduced stromal signatures. The proteomic analysis revealed enrichment in functional annotations associated with an active tumor immune microenvironment (Figs. 2 and 3). These findings were supported by histopathological results showing a high presence of TILs (Fig. 5A, B) and concordant DNA methylation-based cell-type deconvolution, which revealed high infiltration of multiple T lymphocyte lineages (Fig. 6A–C). Collectively, these data suggest that in the IMA subtype, effector immune cells in the TME may still be capable of recognizing and eliminating tumor cells. This is evident by the functional annotations describing antigen presentation and regulation of T cell activation, as well as high proportions of the DC, CD4 + T cell, and CD8 + T cell types. More importantly, our study provides evidence for potential mechanisms of immune escape and evasion employed by these tumors. The IMA subtype tumors appear to recruit immune suppressive cells to the TME, based on high proportions of Tregs (Fig. 6C), and activate immune inhibitory cascades, such as the PD-1 signaling pathway (Fig. 3A). Defects in priming or incomplete activation of effector cells by DCs in the IMA subtype patients could lead to immune evasion. For example, DCs must prime T cells to initiate an effective anti-tumor immune response. However, missing co-stimulatory signals during the priming process can result in T cell anergy when T cells fail to develop full effector function or functional hyporesponsiveness^46,77^. Furthermore, CD8+ cytotoxic T cells might reach an exhausted state over time due to sustained over-stimulation from prolonged exposure to antigens and chronic inflammation, leading to progressive loss of effector capability. This reduced ability of CD8+ cytotoxic T cells to eliminate target cancer cells ultimately results in immune evasion^78,79^. Additionally, the differential analysis of IMA subtype-specific proteins showed an increased abundance of IDO1 (Supplementary Fig. 7A), an enzyme secreted by cells in the TME to promote immune tolerance and suppression^80,81^. Taken together, these findings suggest that patients with IMA subtype tumors would likely benefit from immune checkpoint inhibitors combined with chemotherapy. These observations are in agreement with other profiling studies of TNBC subtypes^30,33,34,36,38–41^, which have identified an immune-“activated”, immune-“hot” or “immunomodulatory” subtype with similar characteristics, wherein the TNBC subtype with the highest immune presence exhibits the best prognoses (Fig. 5D), and earlier cancer stages at diagnosis (Supplementary Fig. 4C).

The LAR subtype features an enrichment of metabolic signatures and low enrichment in cell cycle and RNA processes. In the proteomic analysis, the tumors displayed increased metabolic activity such as fatty acid (FA) β-oxidation, glycolysis, and cholesterol biosynthesis among the over-represented annotations, which could suggest a higher degree of metabolic flexibility that allows the cancer cells to adapt and persist in harsh, nutrient-depleted environments (Figs. 2 and 3). Apart from providing critical biosynthetic precursors and an alternate source of ATP, the oxidation of FAs is a major contributor to ROS production in the cell^82^. Indeed, we observed elevated oxidoreductase activity and ROS-related terms over-represented in the LAR subtype functional annotations. Using immune cell-type proportions inferred from genome-wide DNA methylation profiles, we were surprised to discover that the LAR subtype had high proportions of DC, CD8 + T, B, and NK cell types (Fig. 6A/G-I). Increased ROS production within the TME can create an immunosuppressive milieu, which inhibits the function of immune cells such as T cells and NK cells^53,54^. Specifically, ROS can lead to T-cell exhaustion or anergy, yielding cells with reduced proliferation capacity and functional impairments, such as diminished cytokine production and responses to tumor antigens^55^. This mechanism may explain how, with such high levels of DCs and CD8 + T cells in the TME, LAR tumors implement immune evasion. The clinicopathological assessment revealed phenotypes similar to those observed in other molecular profiling studies that have isolated LAR as one of the main TNBC subtypes^30,33,34,40^. LAR subtype patients were significantly older, with their tumors exhibiting the lowest abundance of Ki67 protein^36^ (Fig. 4A, D). They also displayed characteristic apocrine differentiation and high AR Allred scores (Supplementary Fig. 4D and Fig. 4C). Despite exhibiting stronger AR positivity compared to the other subtypes via IHC, treatment with anti-AR agents has shown little efficacy in clinical trials^83–85^. Thus, our observation that the representative LAR cell lines were sensitive to mTORC1/2 inhibitor (AZD8055) and p38/JNK inhibitor (Doramapimod) warrants further exploration (Fig. 7C, D). Only recently have limited targeted therapies been approved to treat TNBC in the metastatic setting. One such therapy, sacituzumab govitecan, selectively targets tumor cells expressing Trop-2 protein^6^. Intriguingly, our proteomics analysis revealed that the LAR subtype has the highest level of Trop-2 protein across the subtypes, which offers the potential as a much-needed targeted therapy option for this subtype (Supplementary Fig. 7B).

Although the IMS subtype exhibits high metabolic, DNA repair, and RNA biology related signatures, its most prominent feature is its low immune signature, with previous studies going so far as to call the subtype an “immune desert”^86,87^. Proteomics analysis resulted in the under-representation of immune-related terms, suggesting that many of the major immune cell types, including T lymphocytes, both cytotoxic and helper, B lymphocytes, DCs, neutrophils, and NK cells, are sparse in the TME (Figs. 2 and 3). In concordance with the functional analysis, the TIL scoring and the cell-type deconvolution also showed a low abundance of immune cells in the TME (Figs. 5B and 6). In addition, the cell-type deconvolution analysis revealed the highest proportion of stromal, endothelial, and epithelial cells among the subtypes, indicating the possibility of high CAF presence. CAFs are known to promote metabolic reprogramming, immunosuppression, and therapeutic resistance^88,89^, in part due to the secretion of the immunomodulator, TGFβ2^88,90,91^, a protein that was significantly elevated in the IMS subtype (Supplementary Fig. 7c). Upon histopathological assessment, the IMS subtype tumors exhibited the second largest average tumor size, second highest proportion of lymphovascular invasion (50%), late-stage disease (27%), and tumors with SBR grade 9 (92%) (Fig. 4B and Supplementary Fig. 4B, C, G). When coupled with an enrichment in cell cycle- and proliferation-related functional terms, as well as the highest level of Ki67 protein abundance, these data suggest a relatively fast-growing and rapidly progressing tumor phenotype.

In our study, the “claudin-low” tumors fell within the IMS subtype, signifying a de-differentiated state (Supplementary Fig. 7D)^92,93^. Mounting evidence reveals immune-suppressed tumors with poor differentiation are harder to treat and demonstrate resistance to therapy, leading to unfavorable prognoses and outcomes^34,88^. Thus, the key to treating this challenging subtype is to expose its underlying resistance mechanisms. As the subtype with the fastest-growing tumors, IMS cancer cells have a high energy demand to meet. Similarly to the LAR subtype, IMS tumors displayed increased metabolic activity, with over-represented functional annotations such as glycolysis and oxidation-reduction processes. To protect against the toxic effects of ROS overproduction that accompanies increased ATP production, cancer cells upregulate antioxidant defense mechanisms to maintain reduction-oxidation (redox) balance, reducing oxidative damage and avoiding apoptosis^82^. Despite having relatively few highly abundant proteins (FC >0.7), the IMS subtype featured increased enrichment of glutathione S-transferase enzymes (Supplementary Table 2). This proposes glutathione conjugation as a resistance mechanism utilized for detoxification of ROS in this subtype and may be exploitable for anti-cancer therapy^94–96^. Furthermore, emerging evidence reports that deregulation of fundamental RNA biology can lead to tumor development and progression^48,49^. The IMS functional annotations showed high enrichment of ncRNA metabolism, tRNA modification, MYC variants, and RNA Polymerase I and III, which may point to potential tumor adaptations that enable resistance against conventional therapies and thereby also warrants further research (Figs. 2 and 3).

The prominent features of the MES subtype are its elevated stromal and immune signatures, alongside reduced metabolic signatures. Proteomic analysis revealed enrichment in EMT, angiogenesis, and ECM remodeling, as well as enrichment in inflammation, humoral immune response, and innate immune system (Figs. 2 and 3). The cell-type deconvolution analysis supported these findings, revealing a diverse TME with high proportions of angiogenic-category cell types, including stromal and endothelial cells (Fig. 6D, E) and select immune-category cell types, such as innate (neutrophils and NK cells) and suppressive cells (Tregs) in the MES subtype (Fig. 6C, I, J). The histopathological assessment showed that the MES subtype had the highest proportion of tumors with prominent fibrosis, consistent with the functional annotation results that implied ECM remodeling, such as collagen assembly, intermediate filament cytoskeletal organization, degradation of ECM, platelet aggregation, and endopeptidase activity (Supplementary Fig. 4H and Fig. 3). Although the analysis exhibited evidence of immune cell infiltration, current TIL scoring guidelines do not count polymorphonuclear leukocytes, such as neutrophils. This might explain the lower TIL levels observed in the MES subtype compared to the IMA subtype (Fig. 5B) despite our other analyses suggesting substantial immune cell infiltration (Figs. 3 and 6J).

The lack of enriched Hallmarks associated with metabolic activity and the pronounced enrichment of EMT and angiogenesis in this subtype suggests that the MES tumors are not as energetically demanding as other subtypes and do not prioritize localized tumor growth (Fig. 2). Rather, the results emphasize more aggressive mechanisms that enable lineage plasticity and promote a conducive microenvironment for invasion and metastasis. To meet the high demands of cancer progression and ECM remodeling, cells stimulate angiogenesis, or the sprouting of new blood vessels from preexisting ones, through the release of VEGF. Thus, the various stromal constituents receiving these signals will remodel the TME to support new vascular growth^97^. Indeed, patients with TNBC exhibit significantly higher levels of VEGF than other BC subtypes^98^. Within TNBC, it is chiefly the MES subtype that has such a microenvironment, and based on various functional annotations, such as VEGF signaling and metabolism of angiotensinogen to angiotensins, patients in this subtype may benefit from angiogenic inhibitors like bevacizumab (Fig. 3). As the earliest approved targeted therapy for TNBC in the metastatic setting^10^, bevacizumab is currently being tested in NCI clinical trials in combination with immune checkpoint inhibitors, for aiding in immune response activation, making the MES group of TNBC patients good candidates for these trials^99–103^. Eribulin may be another viable targeted therapy option for MES subtype patients. As an inhibitor of microtubule dynamics, eribulin exerts its cytotoxic effects on cancer cells by disrupting cell division. However, it has shown additional benefits, such as anti-angiogenic and immunomodulatory properties^104^. Treatment with eribulin leads to suppression of EMT, promoting a morphological transition to a more differentiated epithelial phenotype^105^. Eribulin treatment also results in vascular remodeling, which promotes an increased accumulation of infiltrating immune cells into the TME, inducing a shift from a pro-tumor to an anti-tumor immune environment in responding patients^106–110^. Eribulin was FDA-approved for the treatment of advanced BC patients who are refractory to other forms of treatment^111^. Although these studies reinforce the efficacy of eribulin in BC, within the TNBC subgroup of patients further research is still needed to find a reliable system to predict and stratify responders.

## Methods

### Clinical sample acquisition

A total of 55 TNBC specimens were obtained from DHMC institutional archives based on the availability of sufficient primary tumor tissue. Patients were selected from 2003 to 2015. Hematoxylin and eosin (H&E) stained sections were reviewed by a breast pathologist (JDM) to select the FFPE blocks with sufficient tumor cellularity. The following clinicopathological characteristics were collected by retrospective review of medical records and pathology reports: age at diagnosis; pathologic stage at diagnosis; tumor size; lymph node status; Scarff-Bloom-Richardson (SBR) score; the presence or absence of associated DCIS; lymphovascular invasion; ER, PR, and HER2 status; and survival. Survival data cut-off was August 2023.

### Tumor proteomic MS analysis

For proteomic analysis, 10 µm FFPE tumor sections were macro-dissected based on markings from DH Pathology to enrich for malignant cells ( >70% malignant cellularity), deparaffinized by incubation with xylene for 5 min, and rehydrated with three gradient ethanol washes (100%, 96%, and 70%) followed by de-crosslinking buffer (0.1M Tris-HCL, pH 8; 5 mM dithiothreitol (DTT)). Samples were sonicated on ice, and SDS was added to a final concentration of 4%. Samples were heated to 99 °C for 60 min, cooled, and protein was acetone extracted and resuspended in lysis buffer (8 M urea, 75 mM NaCl, 1mM EDTA, 50 mM Tris HCl (pH 8), 2 mM β glycerophosphate, 2 mM sodium fluoride, 2 mM sodium molybdate, 2 mM sodium orthovanadate and EDTA-free Mini-Complete protease inhibitors). Proteins were reduced with 5 mM DTT for 30 min at 55 °C, cooled to room temperature (RT), alkylated with 20 mM iodoacetamide at RT in the dark for 1 h, and quenched with 5 mM DTT. Proteins were digested using trypsin (1:100 w/w) into peptides, desalted, and labeled with tandem mass tag (TMT) reagents. Labeling efficiency was confirmed to be >95%. Tumors were randomly assigned into six separate TMT-11-multiplexes. Samples were pooled, each multiplex was offline-fractionated fractionated by pentafluorophenyl (PFP)-based reversed phase chromatography^112^, and then analyzed by LC-MS.

### LC-MS/MS sample preparation and analysis

Peptides were desalted on an Oasis desalting plate, then resuspended in loading buffer (5% methanol, 1.5% formic acid) and run on an analytical resolving column (made in house), in-line with an EASY-nLC 1000 ultra-high pressure liquid chromatography. Samples were analyzed on an Orbitrap Fusion Tribrid mass spectrometer and raw data searched using COMET^113^ software against a target-decoy (reversed) version of the human proteome sequence database^114^, and quantified.

### Tumor proteomic computational analysis

The tumor proteomic output files were structured as six TMT-11-plex experiments, which were compiled into one dataset. Intensities for protein abundance data were log_2_ transformed. A frequency of observation cutoff of 93% was implemented (retaining all proteins with at least 53 out of 57 samples with quantified values), resulting in a total of 6,306 proteins in the tumor proteomic dataset. The remaining processing steps were carried out using Perseus software^115,116^ (v2.0.3.0). For missing data imputation, we generated random sampling from a normal distribution, with a downshift of 1.8 and width of 0.3. The data were then normalized by quantile normalization. Finally, the batch effects were estimated and corrected using the ComBat method^117,118^.

### Unsupervised hierarchical clustering

The R package “CancerSubtypes” (v1.24.0)^119^ was used to identify the molecular TNBC subtype clusters from the tumor proteomic data. To visualize the clustering of the tumor proteomic dataset, we used the feature selection method based on variance (SD) from the mean, and the ‘FSbyVar’ function, where the top 1000 most variant proteins in the dataset were extracted. The unsupervised hierarchical clustering analysis was then performed using the ‘aheatmap’ function, with the method for distance measure was specified as kendall and the clustering linkage as ward. The resulting heatmap displayed clear separation of four distinct clusters, and cluster assignments (subtypes) were generated by cutting the resulting dendrogram.

### Principle component analysis (PCA)

Additionally, we applied PCA to visualize the proteomics data in two-dimensional space and to identify possible tumor groups based on proteome-wide similarities^120^. For the PCA, the input data selected was the same input used for the unsupervised hierarchical clustering analysis, which included the top 1000 most variant proteins across all samples in the tumor dataset. The top principal components and their degree of variation were analyzed using the ‘prcomp’ function in the “stats” R package (v4.2.3). The first two dimensions (PC1 and PC2) were plotted, with point colors based on the heatmap dendrogram’s cluster assignments, generated from the unsupervised hierarchical clustering, wherein the PC1/PC2 plane shows well-defined regions that re-identify the separate subtypes.

### k-means consensus clustering

Consensus k-means clustering was performed to independently assess the optimal number of TNBC subtypes in the tumor proteomic data. For unsupervised consensus clustering analysis, we selected the top 75% most variable proteins based on median absolute deviations (MAD). The R package “ConsensusClusterPlus”(v1.62.0)^121^ was implemented using the Partitioning Around Medoids (PAM) clustering algorithm with maximum distance metric. A subsampling parameter of 80% with 1000 iterations was applied, and a number of potential clusters (k) ranging from 2 to 10 was assessed. The optimal number of k clusters was determined to be four based on 1) visual inspection of the consensus matrices, 2) the shape of the cumulative density function (CDF) distribution plot, and 3) the delta plot displaying the relative change in the area under the CDF curve.

### Functional annotation analyses

Differentially abundant proteins (DAPs) were defined as proteins showing abundance values that were significantly higher or lower in one subtype compared to the others. The statistically significant p-value threshold was set at < 0.05, proteins were arranged by log_2_-fold change (FC), and the FC threshold was set at the 200 lowest and highest DAPs in each subtype. Functional annotation analyses were performed in WebGestalt^44^ using the subtype-specific lists of DAPs as input. We searched KEGG (Release 88.2)^122^, Reactome (Version 66, September 2018)^123^, Wikipathways (Release 02/10/2020)^124^, and PANTHER18.0 (v3.6.1, 01/22/2018)^125,126^ pathways databases, as well as Gene Ontology (GO)^127,128^ and Hallmark 50^129^ gene set annotations separately. The Benjamini–Hochberg (BH) method was used to account for multiple hypothesis testing, and for the remaining options default settings were used. The resulting enrichment ratio scores, p-values, and FDR scores were recorded and utilized to indicate that a category is over-/under-represented. Graphical visualizations of the resulting functional annotations were simplified based on biological relevance and generated using GraphPad Prism (v10.1).

### Application to dataset from Anurag et al.^35^

A total of 886 proteins of the 1000 most variable proteins identified in this analysis were identified and quantified in Anurag et al.^35^ with a 50% frequency of observation. Missing proteins were imputed from a random sampling of a normal distribution, with a downshift of 1.8 and width of 0.3. Unsupervised hierarchical clustering analysis was performed using the ‘aheatmap’ function, with the method for distance measure was specified as kendall and the clustering linkage as ward. The resulting heatmap displayed separation of four distinct clusters, and cluster assignments (subtypes) were generated by cutting the resulting dendrogram. DAPs that were significantly higher or lower in one subtype compared to the others were used for functional annotation analyses in WebGestalt^44^ as described above. Graphical visualizations of the resulting functional annotations were simplified based on biological relevance and generated using GraphPad Prism (v10.1).

### Functional annotation grids

Heat map grids showing relevant GO terms and pathways deregulated in particular TNBC tumor subtypes were constructed using GO terms or pathways statistically significant (FDR < 0.05, with BH adjustment) in at least one subtype. Each column represents one of the four proteomic tumor subtypes and rows indicate annotations. The enrichment ratio scores for each subtype were plotted. Higher scores shown in red demonstrate over-represented annotations, lower scores shown in blue demonstrate under-represented annotations, and white blocks indicate a lack of annotations in the specific subtype.

### Cell-type proportion analysis

For DNA methylation-based cell type deconvolution, DNA methylation profiles were obtained from patient samples. Genomic DNA from tumors was isolated using a DNeasy Blood & Tissue Kit (QIAGEN) according to the manufacturer’s protocol. After DNA extraction, quantification, and bisulfite modification, Infinium MethylationEPIC Bead Chips (Illumina, Inc., CA, USA) were used to measure the DNA methylation status of bisulfite-modified DNA samples. Raw probe intensity data, iDAT files, from the methylation array were processed through preprocessNoob using minfi (v. 1.47.0)^130^ R package, and quality control was performed using ENmix (v. 1.26.9) R package. Next, using the lluminaHumanMethylationEPICanno.ilm10b4.hg19 annotation file, 106,522 probes previously reported as cross-reactive, SNP-associated, and non-CpG methylation were excluded. After these exclusions, 759337 CpGs remained for downstream analysis. Beta values were calculated for each CpG using minfi (v. 1.47.0)^131^ R package. Using the tumor beta-values, tumor, angiogenic, and immune cell-type proportions were estimated with HiTIMED (v. 0.99.3)^70^ R package.

For protein-based cell type deconvolution, proteomics data from triple-negative breast cancer (TNBC) samples were deconvolved using the ProteoMixture software. ProteoMixture applies computational deconvolution algorithms to predict the relative contributions of tumor, stromal, and immune cell components within cancer tissue samples based on protein-level abundance data^71^. Protein-level abundance data were log-transformed and normalized prior to deconvolution.

### Estimation of genetic ancestry

Genetic ancestry was estimated based on 59 single nucleotide polymorphism (SNP) genotyping probes from the MethylationEPIC array. The 59 SNP probes are also available on the legacy 450K array, permitting the use of the TCGA data to train and test supervised machine-learning models. The *n* = 768 TCGA breast cancer cases with both methylation and race were split into 80% training and 20% test sets. Three well-characterized supervised models were optimized in parallel: elastic-net logistic regression, random forest, and support vector machines, yielding comparable 5-fold cross-validation and hold-out test performance. The final models re-trained on all TCGA samples with the optimal hyperparameters were applied to the TNBC Cohort. The classifiers generated the same output except for six cases, which might involve multiple ancestries not resolvable by genetic data, hence annotated as “unknown”. The supervised training, evaluation, and application procedures were implemented in scikit-learn (Python 3.9 library version 0.22.1).

### Pathological assessment

All specimens were classified as triple-negative based on immunohistochemical analysis of ER/PR expression and HER2/neu gene amplification by fluorescence in situ hybridization, according to the American Society of Clinical Oncology/College of American Pathologists guidelines^132,133^ H&E-stained sections were reviewed by a breast pathologist (JDM) to evaluate for specific pathologic features: the presence or absence of apocrine cytology (abundant granular eosinophilic cytoplasm, enlarged round nuclei with prominent nucleoli); prominent stromal fibrosis ( >50% of tumor mass composed of fibrosis); and tumor borders (irregular/infiltrating vs, circumscribed/pushing). Other than carcinomas with apocrine differentiation, there were no pure special type TNBCs in this cohort (e.g. metaplastic carcinoma, adenoid cystic carcinoma, etc.). AR immunohistochemistry was performed using clone AR441 (Dako, 1:50) and an Allred score was assigned.

### Evaluation of tumor infiltrating lymphocytes (TILs)

TILs were estimated from pathology slides using internationally established guidelines developed by the International Immuno-Oncology Biomarker Working Group (http://www.tilsinbreastcancer.org/). In brief, the relative proportion of stromal area to tumor area was determined from the pathology slide of a given tumor region. TILs were reported for the stromal compartment ( = percent stromal TILs). The denominator used to determine the percent stromal TILs was the area of stromal tissue (that is, the area occupied by mononuclear inflammatory cells over total intratumoral stromal area) rather than the number of stromal cells (that is, the fraction of total stromal nuclei that represent mononuclear inflammatory cell nuclei). This method has been demonstrated to be reproducible among trained pathologists^134^. The International Immuno-Oncology Biomarker Working Group has developed a freely available training tool to train pathologists for optimal TIL assessment on hematoxylin-eosin slides (http://www.tilsinbreastcancer.org/).

### Survival analyses

Survival analyses were performed using Kaplan–Meier survival curves plotted to compare the overall survival (OS) differences between subtypes. OS was defined as the time from the diagnosis date to death, with 20 years as the endpoint. The log-rank test was used to calculate the statistical significance of survival time differences at the threshold of P-value < 0.05. Kaplan–Meier curves were visualized, and statistical tests were performed in GraphPad Prism.

### Cell line culture and proteomic analysis

The following human TNBC cell lines were used in this study: MDA-MB-453, MDA-MB-468, CAL148, CAL120, CAL51, BT549, HCC1806, HCC1937, HCC1143, HDQP1, CAL148 and MFM-223. Cell lines were obtained from the American Type Culture Collection (ATCC) or the German Collection of Microorganisms and Cell Cultures (DSMZ). Each cell line identity was verified by short tandem repeat profiling, and all cells were maintained according to literature^30^. For MS analysis, cells were lysed in lysis buffer (8 M urea, 75 mM NaCl, 1mM EDTA, 50 mM Tris HCl (pH 8), 2 mM β glycerophosphate, 2 mM sodium fluoride, 2 mM sodium molybdate, 2 mM sodium orthovanadate and EDTA-free Mini-Complete protease inhibitors), reduced and alkylated, trypsin-digested, and labeled with TMT reagents. The peptides from each cell line were assigned one channel of a TMT-11plex, the assignments are as follows: MDAMB453 (126), CAL148 (127N), MFM223 (127 C), HCC1143 (128N), HCC1937 (128C), BT549 (129N), MDAMB468 (129C), CAL120 (130N), CAL51 (130C), HDQP1 (131N), HCC1806 (131C). Once the labeling efficiency was confirmed to be >95%, reactions were quenched, pooled together, and desalted. The pooled multiplex was pre-fractionated off-line by HPLC on a pentafluorophenyl (PFP) column^112^, producing 16 fractions that were then analyzed by LC-MS/MS on an Orbitrap Fusion Lumos Tribrid instrument.

### Cell line proteomic computational analysis

The intensities for protein abundance data in each TMT channel were log_2_ transformed. A frequency of observation cutoff of 64% was implemented (retaining all proteins with at least 7 out of 11 samples showed quantified values. The remaining processing steps, including missing values imputation and quantile normalization, were carried out using Perseus software (v2.0.3.0)^115,116^.

### Cell line clustering analysis

The R package “CancerSubtypes” (v1.24.0)^119^ was used for identifying the molecular TNBC clusters from cell line proteomics data. To visualize the clustering of the cell line proteomic dataset, we used the feature selection method based on median absolute deviations (MADs), and the ‘FSbyMAD’ function, where the top 1,100 most variant proteins in the dataset were extracted. The unsupervised hierarchical clustering analysis was then performed using the ‘aheatmap’ function, with the method for distance measure specified as kendall and the clustering linkage as ward. The resulting heatmap displayed clear separation of three distinct clusters, and the cluster assignments were generated by cutting the resulting dendrogram. For assignment of cell lines to tumor subtypes, differentially abundant proteins (DAPs) with a fold-change of (-0.8 < FC >0.8, p < 0.05) in the cell line dataset and present in the tumor proteomics dataset were selected. Protein abundances were converted to z-scores. Averaged representative tumor subtype protein abundances were computed by taking the median across all samples in each subtype and combined with the cell line dataset. Unsupervised hierarchical clustering and differential abundance analysis were performed.

### Drug sensitivity database analyses

Drug sensitivity profiles of the 11 TNBC cell lines were assessed in the Genomics of Drug Sensitivity in Cancer (GDSC)^135^. Sensitivity and resistance of TNBC cell lines in response to drugs were measured through z-values, with negative values showing sensitivity and positive values showing resistance. The dataset GDSC2 was primarily mined and each cell line’s z-score (representing its sensitivity to a particular drug) was graphed relative to each other.

### Statistical analyses and software suites

Methods for statistical analyses are mostly described and referenced in their respective method subsections. Software used to perform statistical analyses included: R software (v4.2.3), GraphPad Prism (v10.1, GraphPad Software, La Jolla California USA), Perseus software package (v2.0.3.0, Max Planck Institute of Biochemistry, Martinsried, Germany), WebGestalt v2019^44^. A two-sided Student’s *t* test was applied to normalized protein abundances for differential abundance analysis. Pairwise comparisons, comparing two independent groups, such as tumor size between two subtypes, were computed using the Mann–Whitney *U* test. When comparing three or more independent groups, the Kruskal–Wallis test was used for non-parametric data such as TIL scores among subtypes, and an ANOVA test for normally distributed data, such as immune cell proportions among subtypes. For categorical variables versus categorical variables, such as the comparisons of clinicopathologic factors, Fisher’s exact test was used. Kaplan-Meier curves and log-rank methods were employed for survival analyses. For all statistical analyses, *p* < 0.05 was considered a statistically significant difference. To account for multiple testing, the p values were adjusted to the false discovery rate (FDR) using Benjamini–Hochberg correction.

## Supplementary information

Supplemental materials

Supplemental Table 1

Supplemental Table 2

Supplemental Table 3

Supplemental Table 4

## Data Availability

The raw Illumina MethylationEPIC data is available in Gene Expression Omnibus (GEO) dataset GSE255554, sample accession numbers GSM8074650 through GSM8074707. To review GEO accession GSE255554, go to https://www.ncbi.nlm.nih.gov/geo/query/acc.cgi?acc=GSE255554 and enter token eridwmiipdmxvwv into the box. The raw mass spectrometry data is available at ProteomeXchange (PXD055306) and MassIVE (reviewer password: TNBC!) https://massive.ucsd.edu/ProteoSAFe/dataset.jsp?task=3f0fdbffdefa4dcea091174acc77082f.

## Code availability

Custom code was used in the generation and processing of datasets for this study. The code is implemented in Python 3.9 using scikit-learn (library version 0.22.1). Specific parameters used include the regularization parameter for elastic-net logistic regression, the number of estimators for random forest, and kernel specifications for support vector machines. The code and relevant documentation are available upon reasonable request from the corresponding author, subject to institutional and data-sharing agreements.
