# Supplementary material for: Quantitative proteomics analysis of triple-negative breast cancers
Source: NPJ Precis Oncol. 2025 Apr 24;9:117. doi: 10.1038/s41698-025-00907-8 (PMC12019170; doi:10.1038/s41698-025-00907-8)
Supplement: Supplementary file 1 — Supplemental materials [file 41698_2025_907_MOESM1_ESM.pptx]

## Slide 1
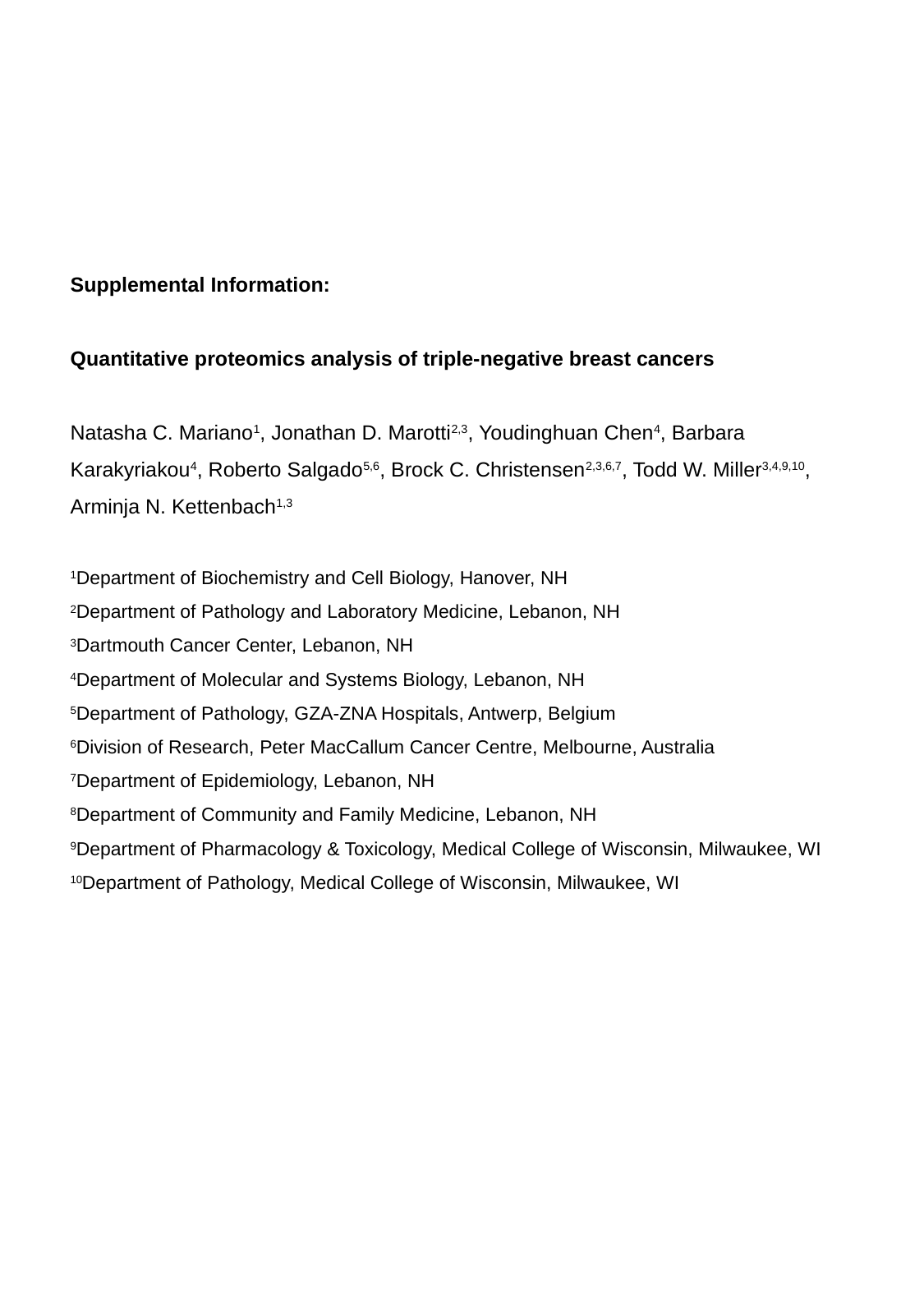

Supplemental Information:
Quantitative proteomics analysis of triple-negative breast cancers
Natasha C. Mariano1, Jonathan D. Marotti2,3, Youdinghuan Chen4, Barbara Karakyriakou4, Roberto Salgado5,6, Brock C. Christensen2,3,6,7, Todd W. Miller3,4,9,10, Arminja N. Kettenbach1,3
1Department of Biochemistry and Cell Biology, Hanover, NH
2Department of Pathology and Laboratory Medicine, Lebanon, NH
3Dartmouth Cancer Center, Lebanon, NH
4Department of Molecular and Systems Biology, Lebanon, NH
5Department of Pathology, GZA-ZNA Hospitals, Antwerp, Belgium
6Division of Research, Peter MacCallum Cancer Centre, Melbourne, Australia
7Department of Epidemiology, Lebanon, NH
8Department of Community and Family Medicine, Lebanon, NH
9Department of Pharmacology & Toxicology, Medical College of Wisconsin, Milwaukee, WI
10Department of Pathology, Medical College of Wisconsin, Milwaukee, WI

## Slide 2
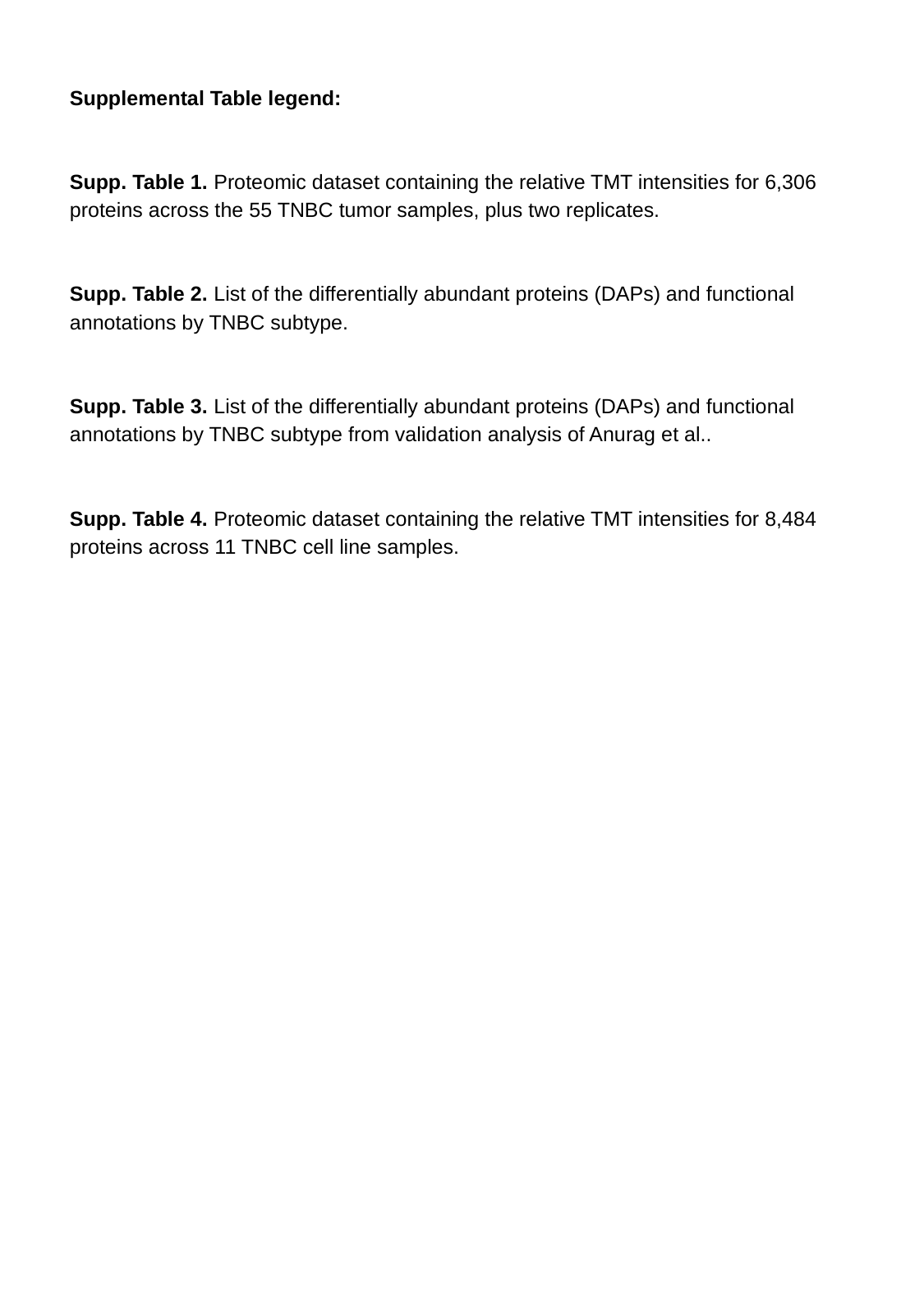

Supplemental Table legend:
Supp. Table 1. Proteomic dataset containing the relative TMT intensities for 6,306 proteins across the 55 TNBC tumor samples, plus two replicates.
Supp. Table 2. List of the differentially abundant proteins (DAPs) and functional annotations by TNBC subtype.
Supp. Table 3. List of the differentially abundant proteins (DAPs) and functional annotations by TNBC subtype from validation analysis of Anurag et al..
Supp. Table 4. Proteomic dataset containing the relative TMT intensities for 8,484 proteins across 11 TNBC cell line samples.

## Slide 3
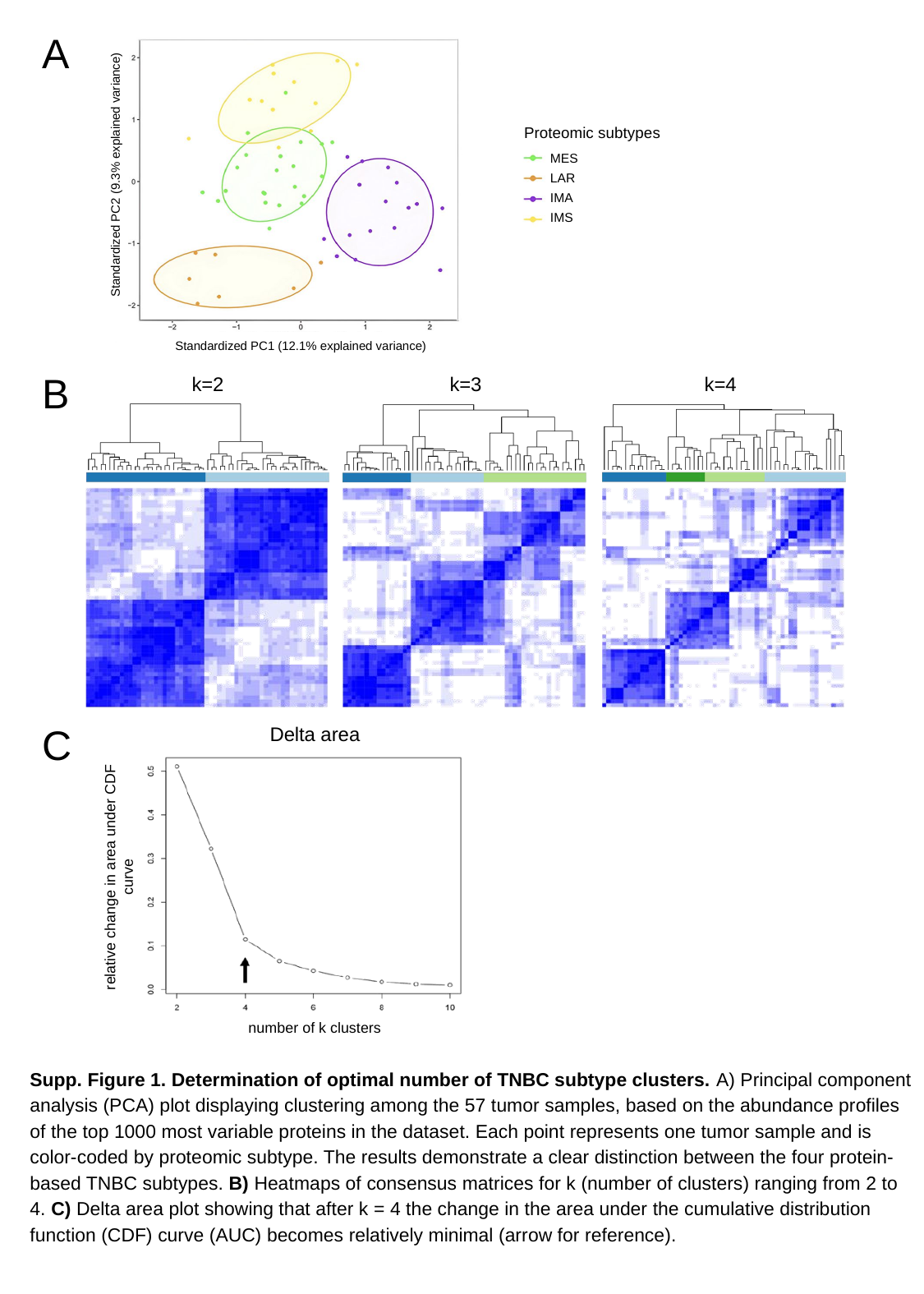

A
Proteomic subtypes
MES
LAR
IMA
IMS
Standardized PC2 (9.3% explained variance)
Standardized PC1 (12.1% explained variance)
B
k=2
k=3
k=4
C
Delta area
relative change in area under CDF curve
number of k clusters
Supp. Figure 1. Determination of optimal number of TNBC subtype clusters. A) Principal component analysis (PCA) plot displaying clustering among the 57 tumor samples, based on the abundance profiles of the top 1000 most variable proteins in the dataset. Each point represents one tumor sample and is color-coded by proteomic subtype. The results demonstrate a clear distinction between the four protein-based TNBC subtypes. B) Heatmaps of consensus matrices for k (number of clusters) ranging from 2 to 4. C) Delta area plot showing that after k = 4 the change in the area under the cumulative distribution function (CDF) curve (AUC) becomes relatively minimal (arrow for reference).

## Slide 4
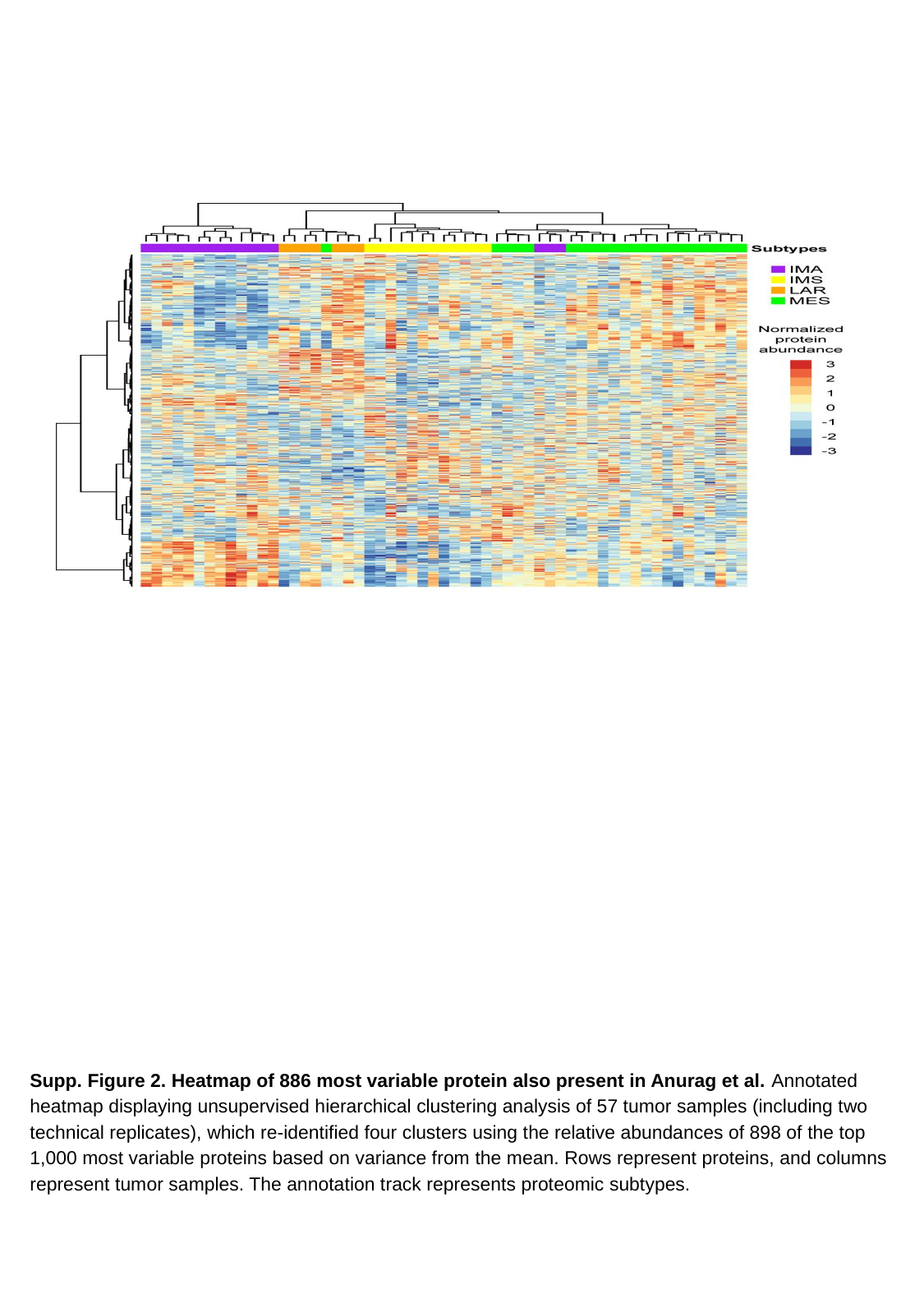

Supp. Figure 2. Heatmap of 886 most variable protein also present in Anurag et al. Annotated heatmap displaying unsupervised hierarchical clustering analysis of 57 tumor samples (including two technical replicates), which re-identified four clusters using the relative abundances of 898 of the top 1,000 most variable proteins based on variance from the mean. Rows represent proteins, and columns represent tumor samples. The annotation track represents proteomic subtypes.

## Slide 5
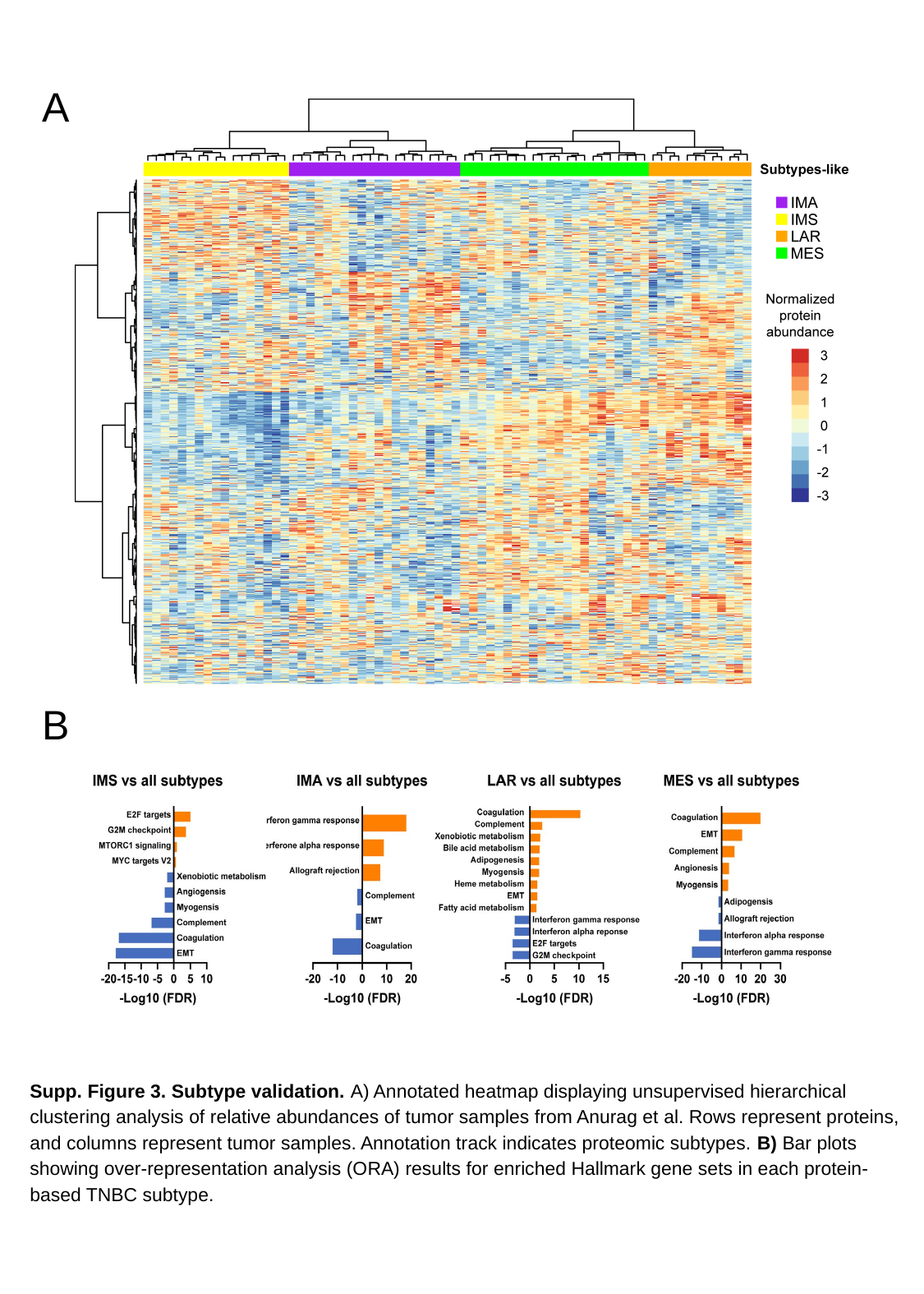

A
B
Supp. Figure 3. Subtype validation. A) Annotated heatmap displaying unsupervised hierarchical clustering analysis of relative abundances of tumor samples from Anurag et al. Rows represent proteins, and columns represent tumor samples. Annotation track indicates proteomic subtypes. B) Bar plots showing over-representation analysis (ORA) results for enriched Hallmark gene sets in each protein-based TNBC subtype.

## Slide 6
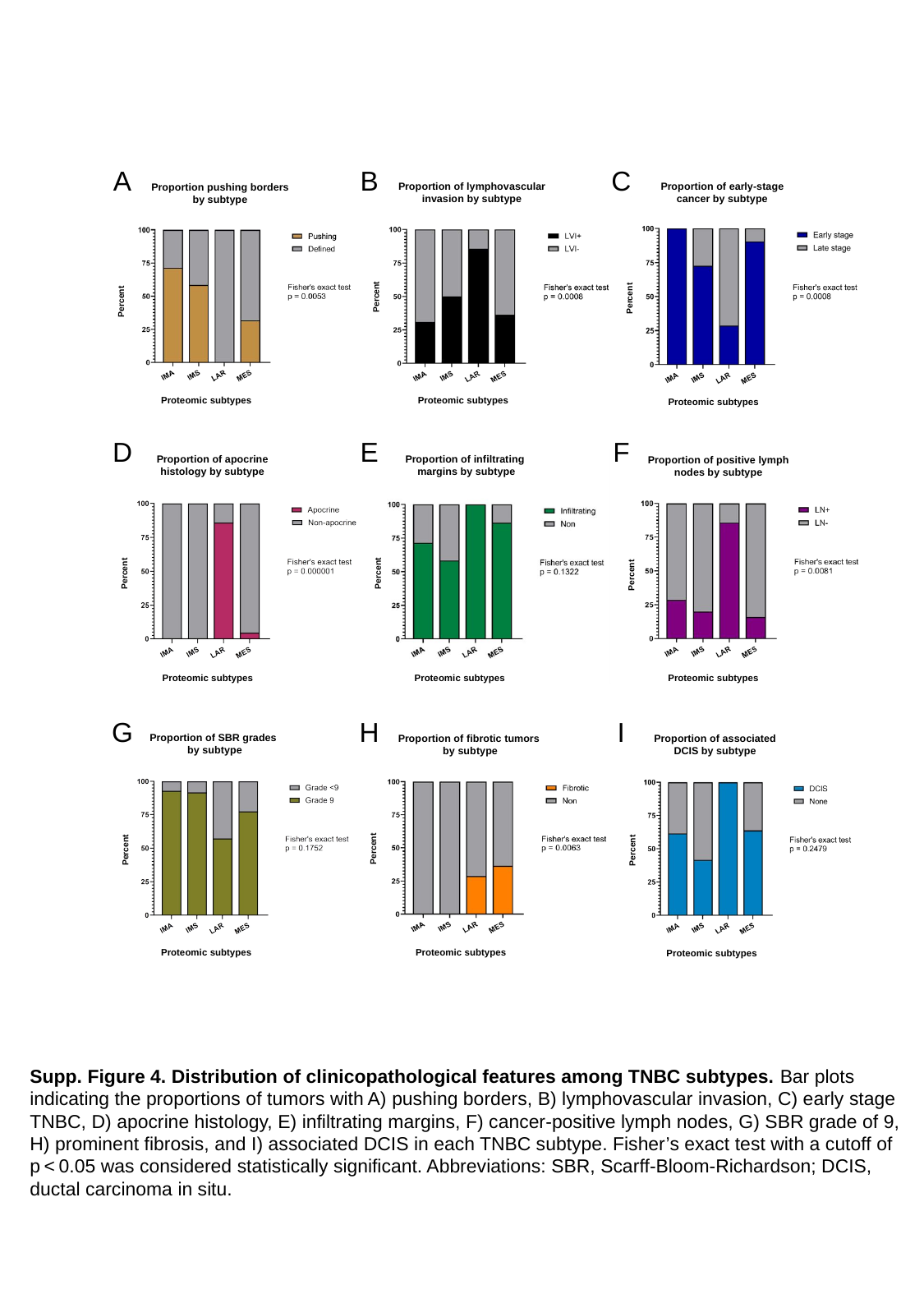

A
B
C
Proportion of lymphovascular invasion by subtype
Proportion of early-stage cancer by subtype
Proportion pushing borders by subtype
Percent
Percent
Percent
Proteomic subtypes
Proteomic subtypes
Proteomic subtypes
D
E
F
Proportion of apocrine histology by subtype
Proportion of infiltrating
margins by subtype
Proportion of positive lymph nodes by subtype
Percent
Percent
Percent
Proteomic subtypes
Proteomic subtypes
Proteomic subtypes
G
H
I
Proportion of SBR grades
by subtype
Proportion of fibrotic tumors
by subtype
Proportion of associated DCIS by subtype
Percent
Percent
Percent
Proteomic subtypes
Proteomic subtypes
Proteomic subtypes
Supp. Figure 4. Distribution of clinicopathological features among TNBC subtypes. Bar plots indicating the proportions of tumors with A) pushing borders, B) lymphovascular invasion, C) early stage TNBC, D) apocrine histology, E) infiltrating margins, F) cancer-positive lymph nodes, G) SBR grade of 9, H) prominent fibrosis, and I) associated DCIS in each TNBC subtype. Fisher’s exact test with a cutoff of p < 0.05 was considered statistically significant. Abbreviations: SBR, Scarff-Bloom-Richardson; DCIS, ductal carcinoma in situ.

## Slide 7
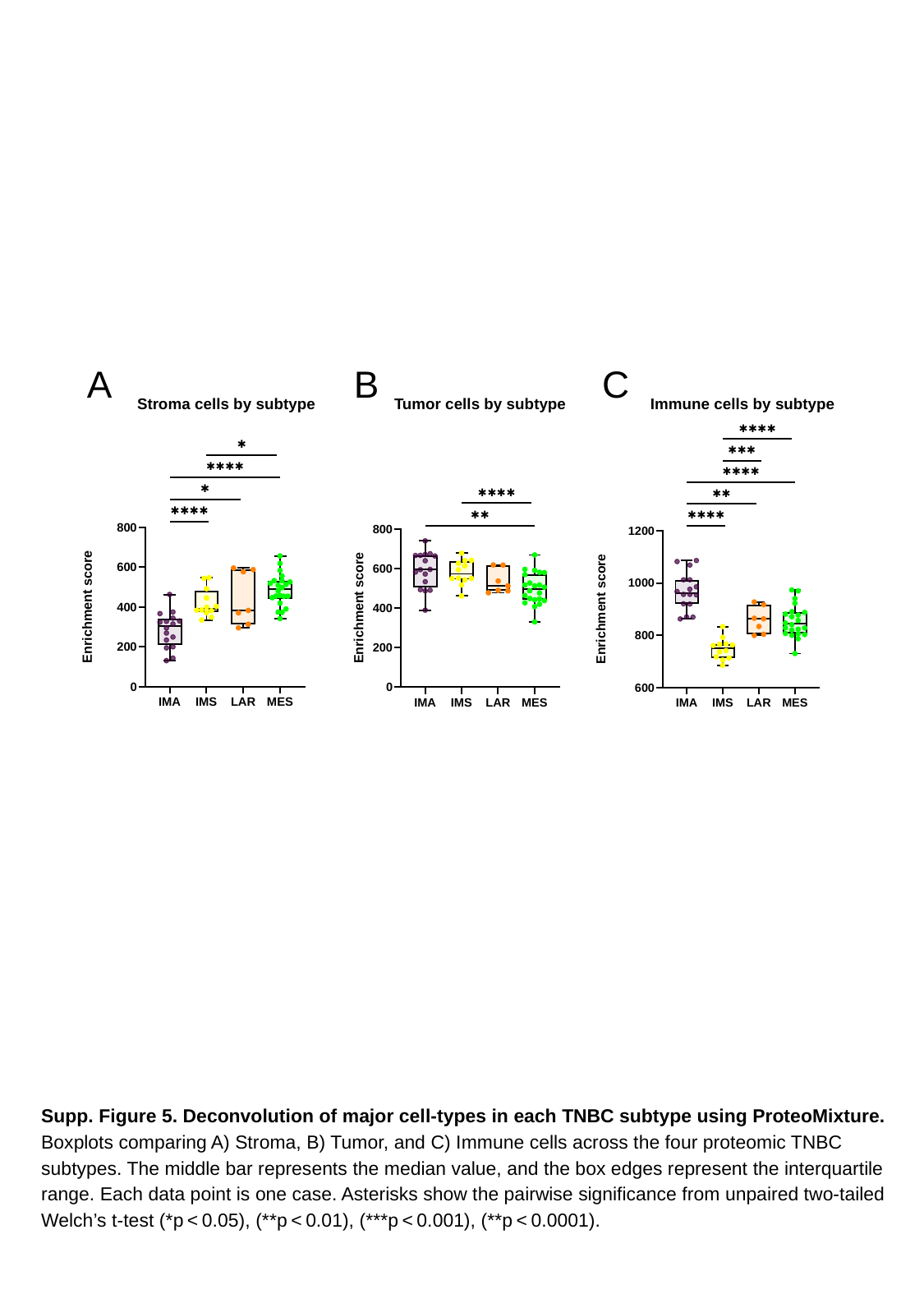

A
B
C
Stroma cells by subtype
Tumor cells by subtype
Immune cells by subtype
Supp. Figure 5. Deconvolution of major cell-types in each TNBC subtype using ProteoMixture. Boxplots comparing A) Stroma, B) Tumor, and C) Immune cells across the four proteomic TNBC subtypes. The middle bar represents the median value, and the box edges represent the interquartile range. Each data point is one case. Asterisks show the pairwise significance from unpaired two-tailed Welch’s t-test (*p < 0.05), (**p < 0.01), (***p < 0.001), (**p < 0.0001).

## Slide 8
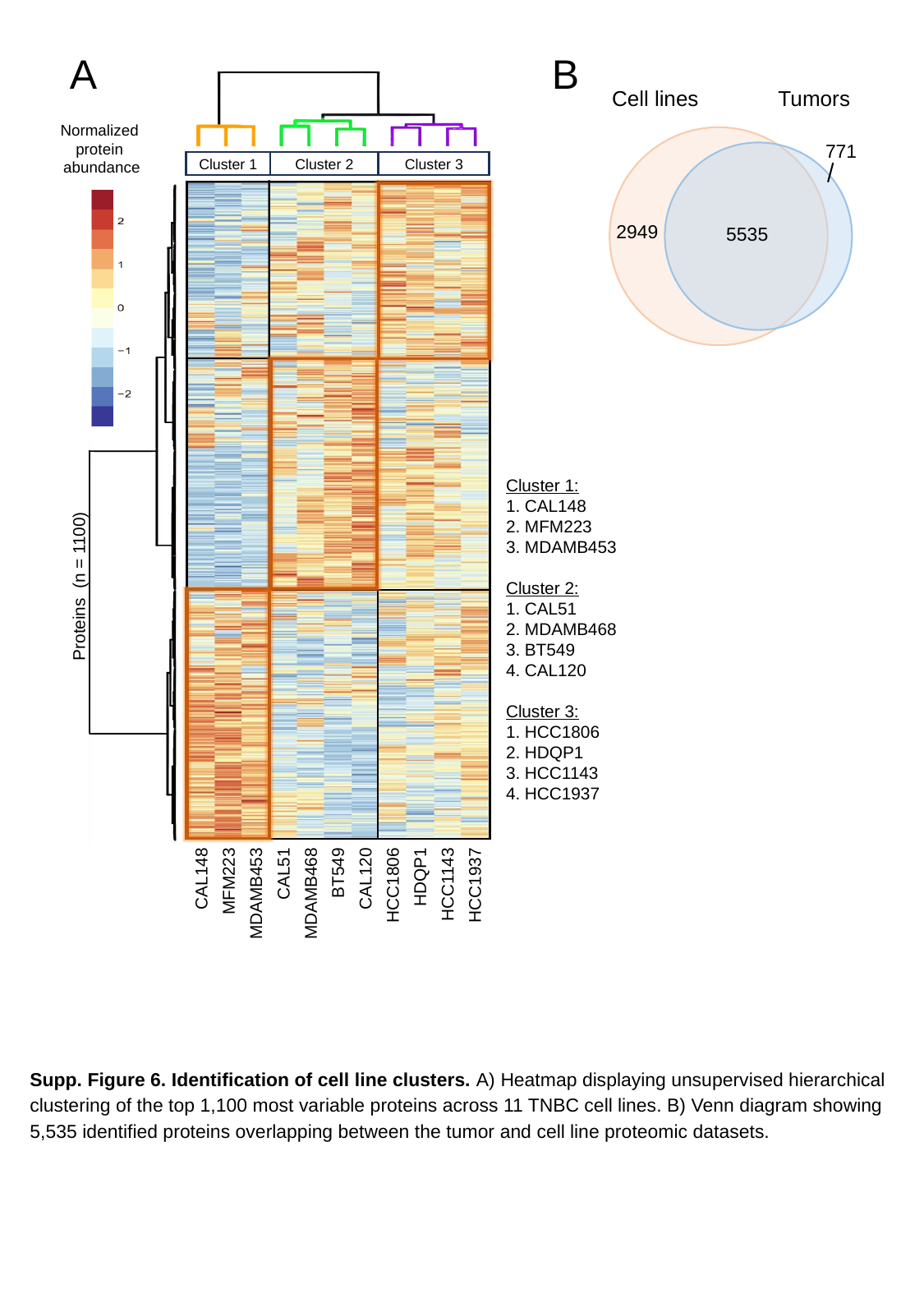

A
B
Cell lines
Tumors
771
2949
5535
Normalized
protein
abundance
Cluster 1
Cluster 2
Cluster 3
Cluster 1:
1. CAL148
2. MFM223
3. MDAMB453
Cluster 2:
1. CAL51
2. MDAMB468
3. BT549
4. CAL120
Cluster 3:
1. HCC1806
2. HDQP1
3. HCC1143
4. HCC1937
Proteins (n = 1100)
CAL148
MFM223
MDAMB453
CAL51
MDAMB468
BT549
CAL120
HCC1806
HDQP1
HCC1143
HCC1937
Supp. Figure 6. Identification of cell line clusters. A) Heatmap displaying unsupervised hierarchical clustering of the top 1,100 most variable proteins across 11 TNBC cell lines. B) Venn diagram showing 5,535 identified proteins overlapping between the tumor and cell line proteomic datasets.

## Slide 9
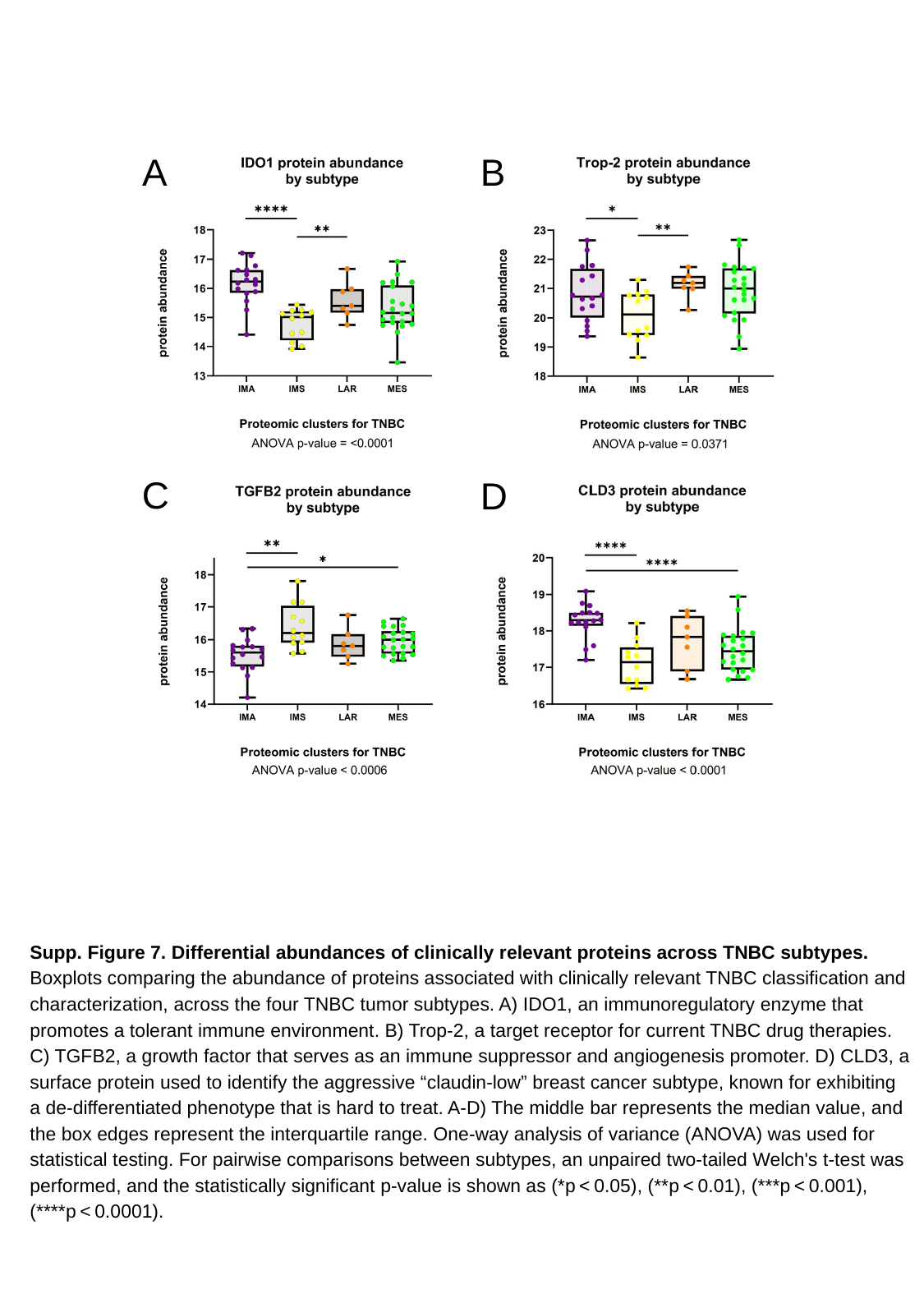

A
B
C
D
Supp. Figure 7. Differential abundances of clinically relevant proteins across TNBC subtypes. Boxplots comparing the abundance of proteins associated with clinically relevant TNBC classification and characterization, across the four TNBC tumor subtypes. A) IDO1, an immunoregulatory enzyme that promotes a tolerant immune environment. B) Trop-2, a target receptor for current TNBC drug therapies. C) TGFB2, a growth factor that serves as an immune suppressor and angiogenesis promoter. D) CLD3, a surface protein used to identify the aggressive “claudin-low” breast cancer subtype, known for exhibiting a de-differentiated phenotype that is hard to treat. A-D) The middle bar represents the median value, and the box edges represent the interquartile range. One-way analysis of variance (ANOVA) was used for statistical testing. For pairwise comparisons between subtypes, an unpaired two-tailed Welch's t-test was performed, and the statistically significant p-value is shown as (*p < 0.05), (**p < 0.01), (***p < 0.001), (****p < 0.0001).
